# Extensive Recombination of a Yeast Diploid Hybrid through Meiotic Reversion

**DOI:** 10.1371/journal.pgen.1005781

**Published:** 2016-02-01

**Authors:** Raphaëlle Laureau, Sophie Loeillet, Francisco Salinas, Anders Bergström, Patricia Legoix-Né, Gianni Liti, Alain Nicolas

**Affiliations:** 1 Institut Curie, PSL Research University, CNRS, UMR 3244, Paris, France; 2 Sorbonne Universités, UPMC Univ Paris 06, CNRS, UMR 3244, Paris, France; 3 Institute of Research on Cancer and Ageing of Nice (IRCAN), CNRS UMR 7284-INSERM U1081, Faculté de Médecine, Université de Nice Sophia Antipolis, Nice, France; 4 Institut Curie, PSL Research University, Next Generation Sequencing Platform, Paris, France; University College Dublin, IRELAND

## Abstract

In somatic cells, recombination between the homologous chromosomes followed by equational segregation leads to loss of heterozygosity events (LOH), allowing the expression of recessive alleles and the production of novel allele combinations that are potentially beneficial upon Darwinian selection. However, inter-homolog recombination in somatic cells is rare, thus reducing potential genetic variation. Here, we explored the property of *S*. *cerevisiae* to enter the meiotic developmental program, induce meiotic Spo11-dependent double-strand breaks genome-wide and return to mitotic growth, a process known as Return To Growth (RTG). Whole genome sequencing of 36 RTG strains derived from the hybrid S288c/SK1 diploid strain demonstrates that the RTGs are *bona fide* diploids with mosaic recombined genome, derived from either parental origin. Individual RTG genome-wide genotypes are comprised of 5 to 87 homozygous regions due to the loss of heterozygous (LOH) events of various lengths, varying between a few nucleotides up to several hundred kilobases. Furthermore, we show that reiteration of the RTG process shows incremental increases of homozygosity. Phenotype/genotype analysis of the RTG strains for the auxotrophic and arsenate resistance traits validates the potential of this procedure of genome diversification to rapidly map complex traits loci (QTLs) in diploid strains without undergoing sexual reproduction.

## Author Summary

The genetic diversity of eukaryotes relies on the diversification of the parental information, mostly occurring by recombination during gamete formation. Homologous chromosomes also recombine in somatic cells, though much less frequently. Here, we sequenced the genome of *S*. *cerevisiae* hybrid diploid cells that enter the processes of meiosis and Return To mitotic Growth (RTG). Remarkably, the RTG cells contain recombined diploid genomes derived from both parental origins. Each RTG cell is diversely recombined both in terms of the frequency and location, with important implications in genome evolution of the species. The generation of a diversely recombined diploid cell population has useful downstream genetic applications.

## Introduction

Genetic diversity relies on diversification of the parental genome information. Besides spontaneous and environmentally induced *de novo* mutations, sexual reproduction is the prominent source of genetic diversity: it reshuffles the genetic information among individuals from a given species, creating the new combinations of alleles upon which the Darwinian selection will potentially act. Thus, the genetic diversity of a given population depends on the random mating of the gametes, the capacity of meiosis to promote homologous recombination between the polymorphic parental chromosomes as well as to ensure the random segregation of the chromosomes in the gametes.

**Fig 1:**
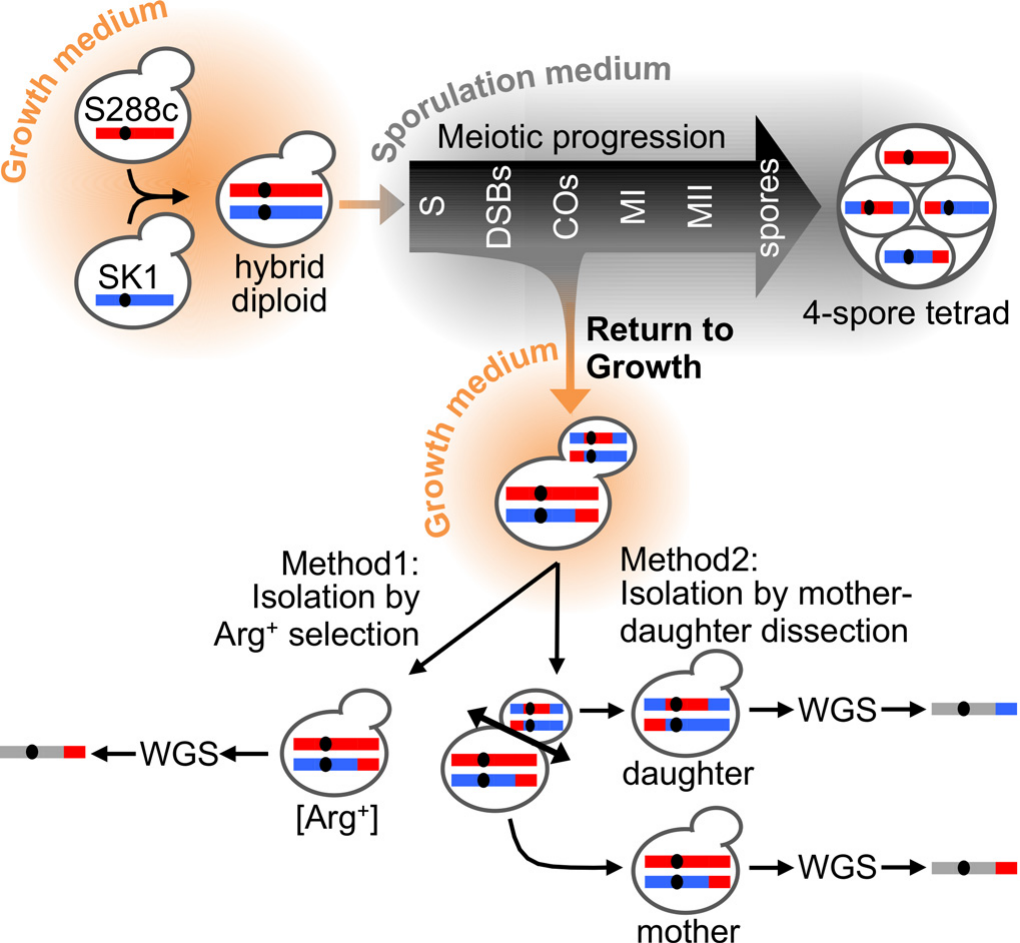
Outline of the landmark meiotic events and methods to isolate RTG cells. The S288c/SK1 hybrid diploid is induced to enter meiosis after transfer into the sporulation medium (1% KAc). The major steps of yeast meiosis are presented on a timeline: S–premeiotic DNA replication, DSBs–Spo11-dependent double strand breaks initiate meiotic recombination, COs–crossover formation, MI–reductional division of the homologs, MII–equational division separating the recombined sister chromatids and formation of four haploid recombinant spores maintained together in a tetrad. The transfer of the meiotic cells into a rich growth medium (YPD) prior to MI reverses the progression of meiosis into a mitotic cell cycle. Return to Growth (RTG), that does not induce replication, allows the transient meiotic mother cell to bud and yield a “daughter” cell. Both the mother and daughter diploid cells inherit two of the four chromatids (recombined or not) that were present in the meiotic cell at the time of RTG induction. Two methods were used to isolate the RTG cells: (left drawing) selection of Arg^+^ recombinant RTG cells generated by intragenic recombination between the *arg4* heteroalleles or (right drawing) separation of the mother and its first daughter cell, a few hours after the induction of the RTG process. Whole Genome Sequencing (WGS) and bioinformatics analyses of the sequencing reads, allows to determine the genotype of the RTG cell at the numerous SNP positions of the parental haplotypes (red: homozygous S288c, blue: homozygous SK1, grey: heterozygous S288c and SK1).

The meiotic developmental program involves the segregation of the homologous pairs of sister chromatids to opposite poles at the first meiotic division (Meiosis-I or reductional division), followed by the segregation of the sister chromatids at the second meiotic division (Meiosis-II or equational division), which is followed by the differentiation of gametes, or spores in yeast (Fig 1)[1]. Another hallmark of meiosis is the high level of inter-homologs recombination during the prophase-I of meiosis. Meiotic recombination is not evenly distributed along the chromosomes but inter-homolog recombination occurs at least once per chromosome [2]. This is initiated by the formation of programmed Spo11-dependent DNA double-strand breaks (DSBs). Afterwards, inter-homolog repair of these DSBs results in the formation of crossovers (CO) and non-crossover (NCO) recombinant products [3]. The relative outcome of CO and NCO events is genetically controlled, depending on the processing of the recombination intermediates and multiple regulatory pathways [4]. Importantly, the crossovers that physically link each pair of homologs ensure the proper reductional segregation at Meiosis-I [5] which ultimately leads to the halving of the genome content and the formation of viable haploid gametes, or spores. Defects in meiotic recombination can arrest the progression of meiosis and are a source of genomic abnormalities and therefore sterility. Notably, the frequent spontaneous formation of disomic chromosome 21 gametes in the male or female gametogenesis is the cause of Down syndrome in humans [6].

In sharp contrast, in all eukaryotes, recombination between the homologous chromosomes is rare in somatic cells [7,8]. Accidental DSBs are preferentially repaired by Non-Homologous End-Joining, a mutagenic process, or repaired in the G2 phase of the cell cycle by homologous recombination between the identical sister chromatids, being promoted by the existence of sister chromatid cohesion that favors recombination between the sisters at the expense of homologs [9,10]. Thus, the rarity of inter-homolog mitotic recombination contributes to the clonal perpetuation of the parental allelic combinations.

Here, to isolate diploid recombinants in yeast, we used the singular, yet remarkable property of *Saccharomyces cerevisiae* diploid cells to exit from the prophase-I of meiosis and be able to re-enter into mitosis, a puzzling process termed “Return to Growth (RTG) [11–14]. As illustrated in Fig 1, budding yeast diploid *MAT***a**/*MAT*α cells are induced to enter meiosis by nutritional starvation [1]; then, the cells enter into S phase and the chromosomes replicate. Next, ~160–200 Spo11-dependent DSBs occur per cell [15] and are efficiently repaired by homologous recombination before the MI reductional division occurs. Remarkably, the highly differentiated and coordinated progression of the DNA intermediates and changes in chromosomal structures through the prophase-I of meiosis is reversible by the addition of rich medium, up to the irreversible commitment point that precedes the reductional division step, thus after the time of DSB formation. This remarkable transition from the meiotic prophase-I to mitosis is under the regulatory control of the Swe1 kinase, which modulates the Cdk1 activity [16]. This kinase permits an unusual progression of the mitotic cell-cycle events, allowing the induction of bud formation in the absence of re-replication [16,17]. Thus, upon RTG, the diploid cell (hereafter called the mother cell) that entered meiosis and experienced DSB formation with or without repair will exit with or without a recombined 4C genome that will segregate equationally, leading to the random segregation of two non-sister chromatids in the mother cell while the other two chromatids will segregate in the daughter cell. Using a single locus intragenic heteroallelic assay, several authors [11,13] observed that the RTG cells were much more frequently recombined than vegetative cells, strongly supporting the conclusion that these recombination events were initiated in meiosis. More recently, physical analyses of the *HIS4-LEU2* hotspot showed that in wild type cells the meiotic Spo11-DSBs are rapidly repaired upon RTG (within 2 h) [17,18] and that the Joint Molecule intermediates (JMs) that accumulate in the prophase-arrested *ndt80Δ* mutant are well repaired upon RTG, but in contrast to meiosis, preferentially give rise to NCO recombinant molecules rather than CO recombinant molecules [17]. Mutant analyses showed that RTG recombination was dependent on the Rad51 strand exchange protein but not Dmc1 [18] and that most JMs are repaired by the Sgs1 pathway that produces only NCOs, while a fraction of JMs are repaired by the Mus81/Mms4 pathway producing both NCOs and COs[17]. But so far however, very little is known about the outcome of the RTG process on the architecture of yeast hybrid genomes.

Here, we report the whole genome sequencing of 36 RTG strains derived from the S288c/SK1 *S*. *cerevisiae* hybrid. We found that the RTG strains are *bona fide* diploids, diversely recombined both in terms of frequency and location. Furthermore, as a proof of principle, we performed a genotype/phenotype analysis of the RTG strains for three Mendelian and one complex traits. This validates the potential of this previously unappreciated procedure of genome diversification to rapidly map quantitative traits loci (QTLs) in diploid strains, without the necessity to undergo sexual reproduction.

## Results

### A yeast hybrid to accurately monitor genome dynamics

To examine the genome-wide recombination profile of RTG cells, we constructed a yeast diploid hybrid (AND1702) by mating two *S*. *cerevisiae* haploid strains from different genetic backgrounds, S288c and SK1 (S1 Table). A similar but differently marked S288c/SK1 hybrid was previously used for meiotic tetrad analyses [19]. Overall, the diploid contains >62,000 single nucleotide polymorphisms (SNPs), distributed along the 16 homologous chromosomes (S1A Fig) resulting in a genome wide divergence of ~0.7% [20,21]. The average inter-SNP distance is 191bp (S1B Fig). Few long regions are devoid of polymorphic SNPs (11 regions ≥10kb). Therefore, this hybrid strain is ideal to achieve high-resolution genotyping and therefore to map recombination events. The strain also carries several auxotrophic markers, appropriate for screening the RTG cells (see below).

The S288c/SK1 hybrid strain sporulates efficiently (88% of asci after 48 h in the sporulation medium), like the SK1 strain and more than the S288c diploid (S2A Fig). However, it produces tetrads with reduced spore viability (71%) relative to both diploid parents (S2B Fig). The distribution of viable spores per tetrad is reported in S2C Fig. The hybrid produces 4 viable spore tetrads (43%) but also a significant fraction of 3, 2, 1 and 0 viable-spores tetrads are observed. Several factors may reduce spore viability [22]. Most likely, an incompatibility between the S288c and SK1 alleles may impair germination and/or growth capacity. In some instances, residual growth observed as micro-colonies are seen under the microscope. An alternative, non-exclusive, hypothesis is the occurrence of Meiosis-I or Meiosis-II chromosome mis-segregations, leading to unviable spores with aneuploid genomes.

### Optimal isolation of RTG cells

In order to isolate the RTG cells, we used two complementary methods illustrated in Fig 1. The first method, “isolation by prototroph selection”, corresponds to the traditional RTG plating assay [11,13] based on the selection of intragenic recombinants, in this case arginine prototrophs (Materials and Methods). The limitation of this selective approach is that upon RTG recombination, only one of the *arg4* alleles is converted to *ARG4* and therefore only the mother or the bud (daughter cell) that inherits the wild type recombinant allele is recovered after RTG. To overcome this limitation, we devised an alternative single cell micromanipulation method to isolate mother cells, that derived from meiosis upon return to growth, from their first daughter cell that arises upon bud formation (Fig 1, Materials and Methods). This micromanipulation method offers two advantages over the prototroph selection. First, it eliminates cells committed to complete meiosis, as they do not form a bud upon transfer to rich medium (they ultimately form tetrads). Second, since re-replication does not occur before budding [16,17], all four chromatids present in the “returned” meiotic cell are recovered in the pair of mother-daughter cells, similar to the recovery of the four products of meiosis in a tetrad. Thus, in RTG pairs, any anomalies in chromosome segregation and marker segregation, including the gene conversion events, can be identified as in four-spores tetrad analyses.

To isolate recombinant RTG cells, the meiotically induced diploid cells should be retrieved at prophase-I of meiosis, after DSB formation and before their commitment to complete meiosis. To determine this time window in the hybrid background, relatively to the SK1 and S288c backgrounds, we monitored and compared several landmark parameters of the meiotic progression in time course experiments. First, we monitored the progression of the cell population during sporulation by DAPI staining of the cell nuclei in the three backgrounds. We observed that the kinetics of meiotic progression of the hybrid resembles more the one of SK1 than of S288c, and that in the hybrid, ~50% completed the Meiosis-I divisions at t = 8 h (S3A Fig). Second, we performed a physical analysis of meiotic DSBs and recombination products formation at the *ARG4/DED81* hotspot of recombination located on chromosome VIII (S3B–S3D Fig). DSB formation at the DSB1 and DSB2 sites [23] is first detected at t = 3 h, similar to SK1 (S3C and S3D Fig). Next, the meiotic recombinant products (R1 and R2) are first observed at t = 5 h and accumulate until 8 h (S3C and S3D Fig). We conclude that, in the cell population, the initiation and completion of meiotic recombination in the hybrid background occur between 4 and 8 hours. Third, we directly examined the occurrence of recombinants in the hybrid by performing a time course RTG experiment. After induction of sporulation for various times in liquid medium, we plated the RTG cells on–Arg plates to select arginine prototroph colonies resulting from intragenic recombination between the *arg4*-*RV* and *arg4-Bgl* heteroalleles [24]. The production of arginine prototrophs arise between t = 4–8 h (S3E Fig), that are enhanced by at least three orders of magnitude, as compared to the basal mitotic level of Arg^+^ colonies observed at t = 0 h. Finally, in the hybrid, since crossovers formed between the heterozygous recessive alleles at *HIS4-LEU2*, *HIS3* and *MET15* loci and their respective centromeres induce loss of heterozygosity (LOH) in RTG (see below), we examined the appearance of auxotrophic colonies in the RTG time course (Materials and Methods). After induction of sporulation for various times, we plated the RTG cells on rich glucose medium and then replica plated the colonies on media lacking histidine, leucine or methionine. Auxotrophic colonies start to appear after 4 h of incubation in the sporulation media and their frequency greatly increases up to 8 h (S3F Fig). Based on these data, the 4–8 hour time window was used for the RTG experiments.

A potential drawback in analyzing a small number of cells from the meiotic time course could be that the cells are not at the expected meiotic stage, due to the relative asynchrony of the meiotic progression. Here, within the 4-8h time window, we observed that an increasing fraction of cells isolated by micromanipulation progressed to give a tetrad (S3G Fig), indicating that those cells had passed the commitment point to irreversibly complete meiosis and sporulation, in a proportion consistent with the kinetics of meiotic progression (S3A Fig). Also, as expected [11–14] [16,17], the vast majority (>94%) of cells that budded gave rise to two viable cells, forming mother and daughter colonies. In the remaining cases, only the mother or daughter cell was viable. Several hypothesis can explain this asymmetric lethality; For example, a technical consequence of separating the mother and daughter cells upon micromanipulation, genetic incompatibilities resulting from loss(es) of heterozygosity in one of the cell or any defect in the process of RTG, independent of the budding process. The rarity of these cases prevented us to further analyze them. In addition, we observed that a significant number of the isolated cells (61% ± 18%, mean ± SD) did not bud nor sporulate (S3G Fig). To eliminate the hypothesis that this lethality is due to the RTG process *per se*, we examined the viability of unbudded cells isolated at earlier time points of meiosis as well as the viability of the vegetative hybrid and SK1 parent cells grown in rich YPD medium and in the pre-sporulation SPS medium. Again, in all cases, a similar proportion of unbudded cells placed on YPD medium by micromanipulation did not grow, indicating that this cell lethality is not meiosis- nor strain-specific (S3G Fig). Other studies have also reported this observation that, after micromanipulation, a high proportion of cells do not divide, especially when cells are isolated from non-logarithmic vegetative culture [25] or from meiotic cultures [26], compared to when cells were isolated from logarithmic vegetative cultures. We do not know the cause of this cell lethality, but in all conditions, the cells that remained on the inoculum area seemed to undergo normal mitotic divisions, suggesting an effect of the micromanipulation. Altogether, we conclude that the meiotic cells that bud after RTG are in most instances viable and as shown below, properly segregate their chromosomes, giving rise to viable euploid cells.

### The RTG cells are extensively and diversely recombined genome-wide

We analyzed 36 RTG strains subjected to high throughput whole genome sequencing (Materials and Methods). Six strains (RTG1-S to RTG6-S) were isolated by Arg^+^ selection (method 1) and 30 strains (RTG7-M/-D to RTG21-M/-D) were isolated by mother-daughter dissection (method 2). Their phenotypes were determined with respect to mating and growth on the Arg, His, Leu and Met depleted media and confirmed by the sequencing data. The genetic marker genotypes are shown in S2 Table. Next, the genotype at SNP positions and recombination profiles were extracted using a dedicated bioinformatic pipeline (S4 Fig) to determine: (i) the chromosome copy number based on coverage depth, (ii) the genotype at SNP positions, requiring the determination of thresholds to call for homozygosity or heterozygosity (S5 Fig), (iii) the extent of LOH, and (iv), the frequency, nature and location of the recombination events in individual strains, using the CrossOver algorithm from the ReCombine program, created for the analysis of tetrad data [27,28].

First, we examined the sequence coverage among the individual chromosomes (S6 Fig and S3 Table). Remarkably, all genomes are euploid, indicating that chromosome segregation in the RTG process was accurate. Nevertheless, two strains (RTG4-S and RTG17-D) displayed a coverage depth variation along two different chromosomes (chromosomes V and XVI for RTG4-S and chromosomes III and V for RTG17-D), revealing in both cases a large duplication and a deletion of over 100 kb (S6 and S7A Figs). The duplication/deletion breakpoints, characterized using the Control-FREEC software [29], are located near the Ty elements of the SK1 chromosomes [30] that are absent in the S288c chromosomes (indicated on S7B Fig for RTG4-S). Molecular validation by Pulsed Field Gel Electrophoresis and Southern blot analysis for the RTG4-S (S7C Fig), suggests that these chromosomal-terminal Gross Chromosomal Rearrangements result from Break Induced Replication initiated between Ty elements located on different chromosomes (S7D Fig).

**Fig 2:**
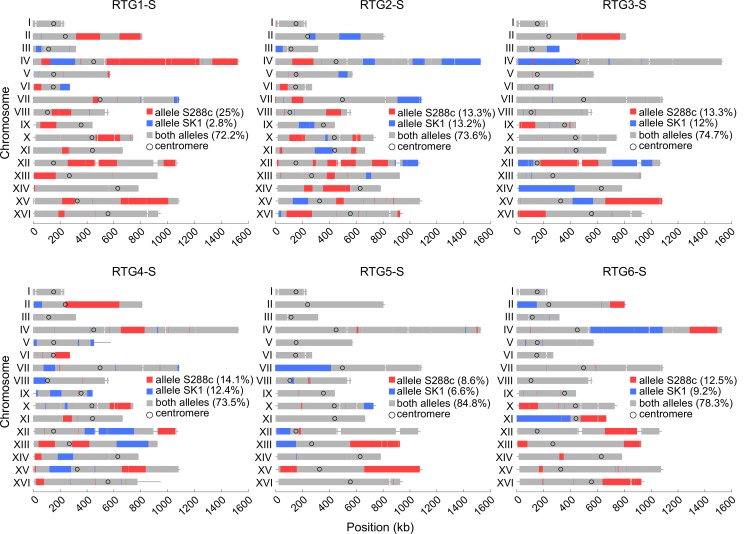
Genome-wide genotype of the six RTG strains obtained upon arginine prototroph selection. The relative frequency of the three genotypes (homozygous S288c in red, homozygous SK1 in blue and heterozygous S288c/SK1 in grey) is indicated in the legend boxes.

The genotype at all SNP positions of the six RTG diploids isolated by selection is shown in Fig 2. In each strain, the vast majority (on average 86.3%) of the SNP positions remained heterozygous as in the parental strain. However, a substantial fraction (on average 13.7%) of SNP positions are homozygous for either parental origin (Fig 2, S4 Table), demonstrating that the RTG strains are recombined. Remarkably, the total amount of polymorphisms exhibiting LOH varies from 15.2 to 27.8% between the RTG strains, demonstrating that the RTG process generates a high degree of genetic diversity.

### Mother and daughter RTG pairs exhibit complementary genotypes

**Fig 3:**
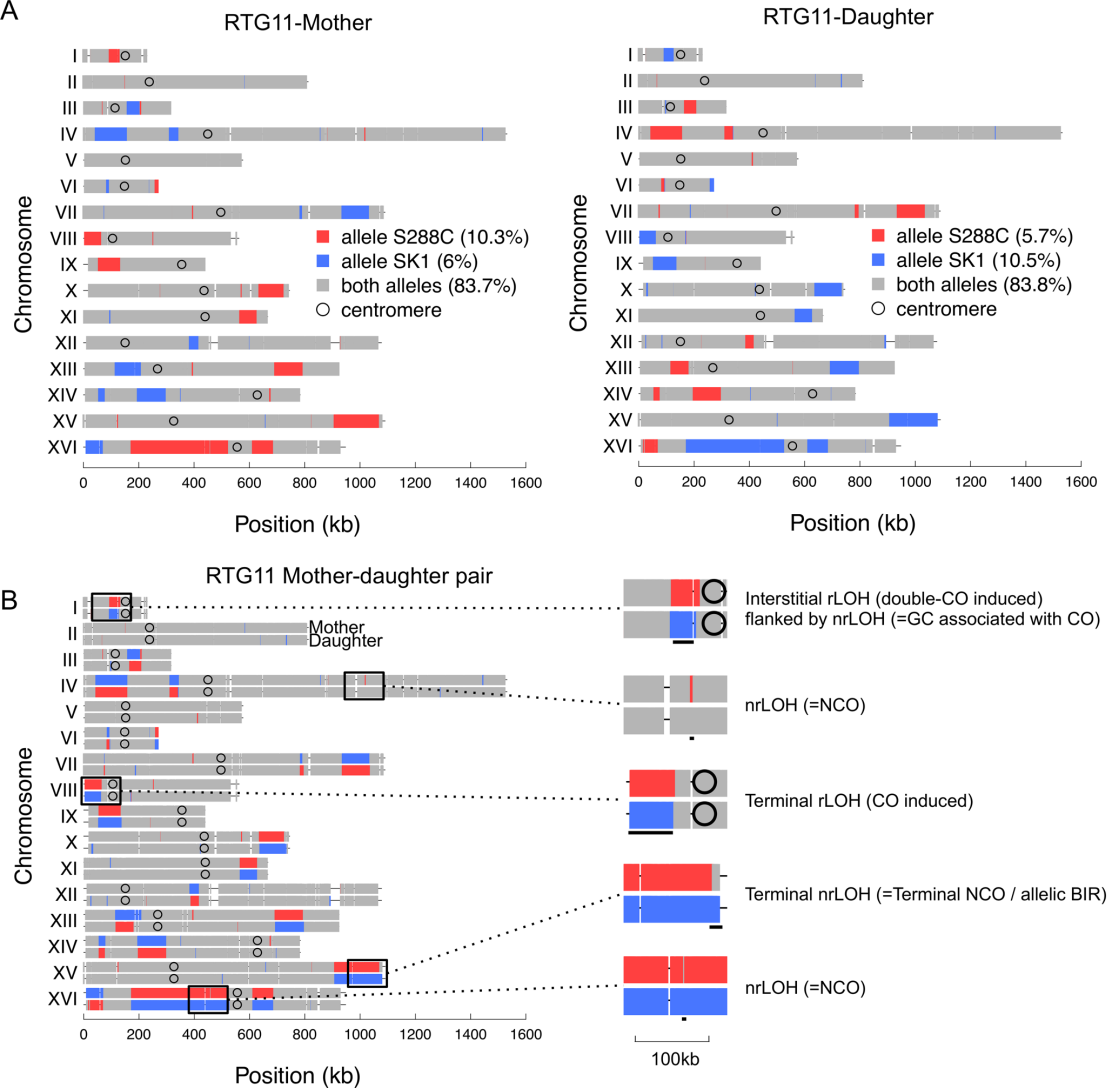
Genotype analysis of the mother and daughter RTG strains. (A) Genotype of the RTG11-M and RTG11-D cells. (B) For comparison, the genotype of the RTG11-M and RTG11-D chromosomes are shown on top of each other, revealing that they have complementary LOH regions. The zoom-ins of particular types of event is shown on the right. Other legends as in Fig 2.

Next, we analyzed the segregation at all SNP positions in the 15 pairs of mother-daughter RTG strains. Since the RTG strains remained diploid, the genotyping of RTG pairs provides tetrad-like information concerning the segregation pattern of the four chromatids derived from of a single meiotic cell that underwent RTG. On average, we observed that for 98.6% of the SNP positions, the genetic information segregated 2:2 in mother and daughter RTG pairs. Among them, 89.2% carry a heterozygous genotype in both mother-daughter cells, as the parent diploid. Conversely, 10.8% of the SNP positions segregating as 2:2 carry a homozygous genotype with opposite parental alleles in the mother and daughter cells (S8 Fig and S4 Table). This is exemplified in Fig 3 in which the genotype of the mother strain (RTG11-M) contains 16.3% of homozygous SNP positions, with 10.3% from S288c and 6% from SK1, while the genotype of the daughter strain (RTG11-D), contains 16.2% of homozygous SNP positions, but with the reverse percentage of parental alleles: 5.7% S288c and 10.3% SK1. The homozygous SNP positions exhibiting a 2:2 segregation pattern, grouped as tracts with reciprocal genotypes, correspond to LOH events resulting from reciprocal exchanges between non-sister chromatids. Thus, the meiotic cell that exits from meiosis (i.e. the mother cell) inherits two non-sister chromatids and the bud (i.e. the daughter cell) inherits the other two non-sister chromatids, as expected from a successful re-entry into mitosis in the absence of DNA replication. The non-sister chromatids are often but not always recombined. As observed for the selected RTG strains (Method 1, see Fig 1), the absolute frequency of acquired homozygosity is very different from one RTG pair to another. In this dataset of 15 RTG pairs, we observed 136 reciprocal LOH tracts (rLOH) (S5 and S6 Tables), with a wide variation, from 1 to 34 tracts per RTG pair. On average, the rLOH tracts are large (141 kb), and in some cases, they cover most of the chromosome arm.

Most of the remaining SNP positions (1.4%) exhibit a 3:1 segregation pattern of the genetic information, and carry a heterozygous genotype in one cell and a homozygous genotype in the other cell. The homozygous SNP positions exhibiting a 3:1 segregation pattern are grouped into tracts with non-reciprocal genotype, corresponding to gene conversion. We found 913 non-reciprocal LOH tracts (nrLOH) (S5 and S7 Tables), from 5 to 139 events per RTG pair, which on average are small (2.3 kb) compared to rLOH. Finally, we also identified a low number of SNP positions that exhibited a 4:0 segregation pattern (0.06%), distributed in 38 tracts (1 to 5 per RTG pair). These events can arise from multi-chromatid gene conversion events or from mitotic recombination events that occurred prior to meiotic induction.

**Fig 4:**
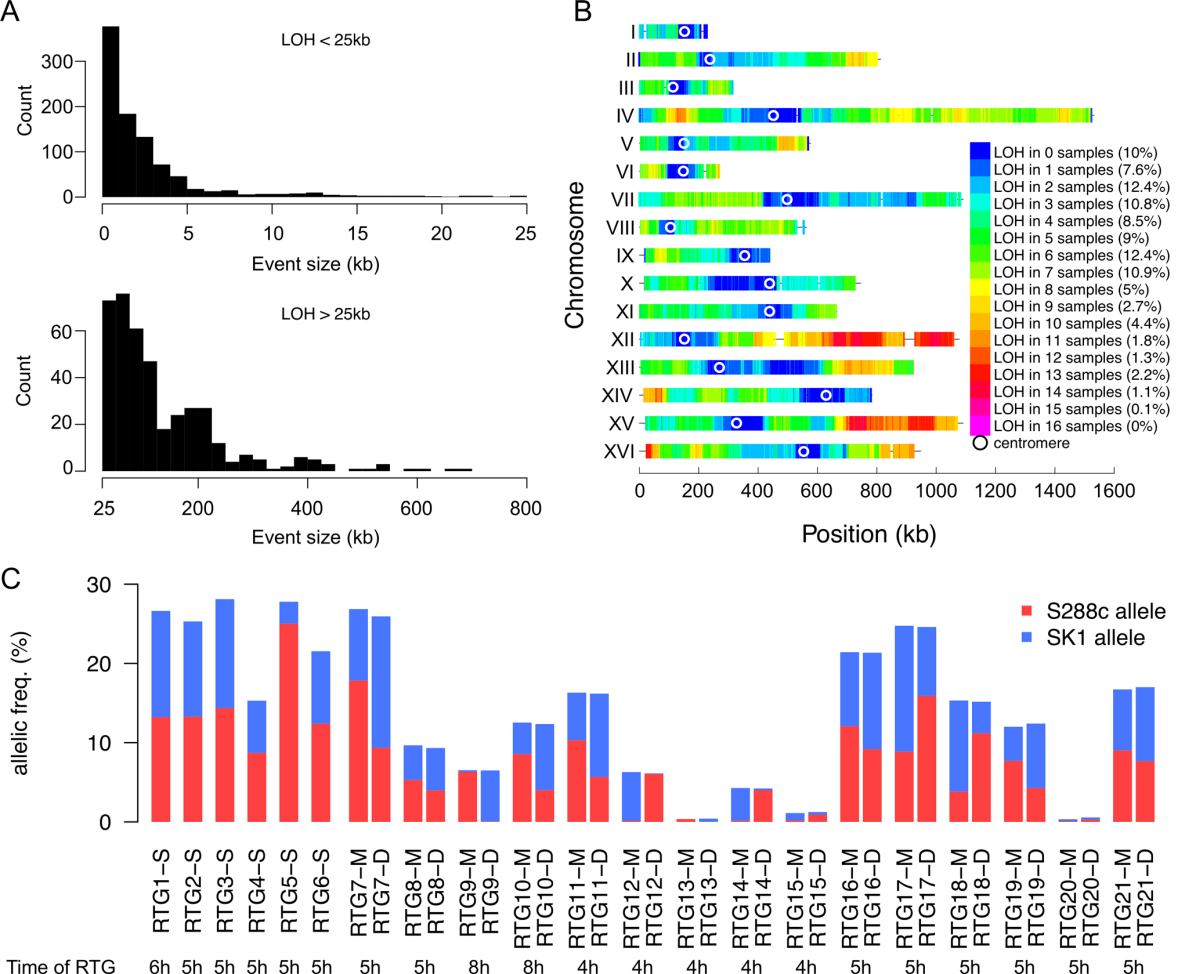
Global genotype analyses of the 36 RTGs. (A) LOH tracts length distribution for the tracts smaller than 25kb (top) or larger than 25kb (bottom). (B) Heat map of the genome wide occurrence of SNP position homozygosity among our 36 RTG strains. Altogether, approximately 90% of the SNP positions were involved in at least one LOH event. The two hotspots (chr. XII-R and chr. XV-R) are likely artificially enriched for homozygosity as they respectively carry the *MET15* and *HIS3* loci that we used to phenotypically screen the RTG cells for auxotrophic phenotypes (Materials and Methods). (C) Homozygous S288c and SK1 allele frequencies.

The number of regions exhibiting LOH ranged from 5 to 87 per RTG strain, with sizes varying between 5 bp to 0.7 Mbp (Fig 4A). Overall, among the 36 RTGs (6 single and 15 mother-daughter pairs), 90% of the SNP positions were involved in at least one LOH event (Fig 4B). On average, 12.2% of the parental hybrid genome shows LOH, ranging from 0.3% (RTG13-M/RTG13-D pair) to 26.4% (RTG7-M/RTG7-D pair). The ratio of parental alleles also varies from one RTG to another (Fig 4C). Among the 10% of SNP positions that failed to exhibit LOH in all RTG strains, the vast majority are located around the 16 centromeres [15,31–34]. Thus, the centromere-linked SNP positions always remain heterozygous after the equational segregation. This is likely attributable to the depletion of meiotic DSB formation and recombination in the vicinity of the centromeres [15,31–34]. To be noted, the maintenance of heterozygosity in the centromere region and in numerous locations along the chromosome arms eliminates the possibility that the LOH event resulted from iso-chromosomal non-disjunctions or reductional division.

### Crossovers and gene conversions contribute to the diversity of the RTG haplotypes

**Fig 5:**
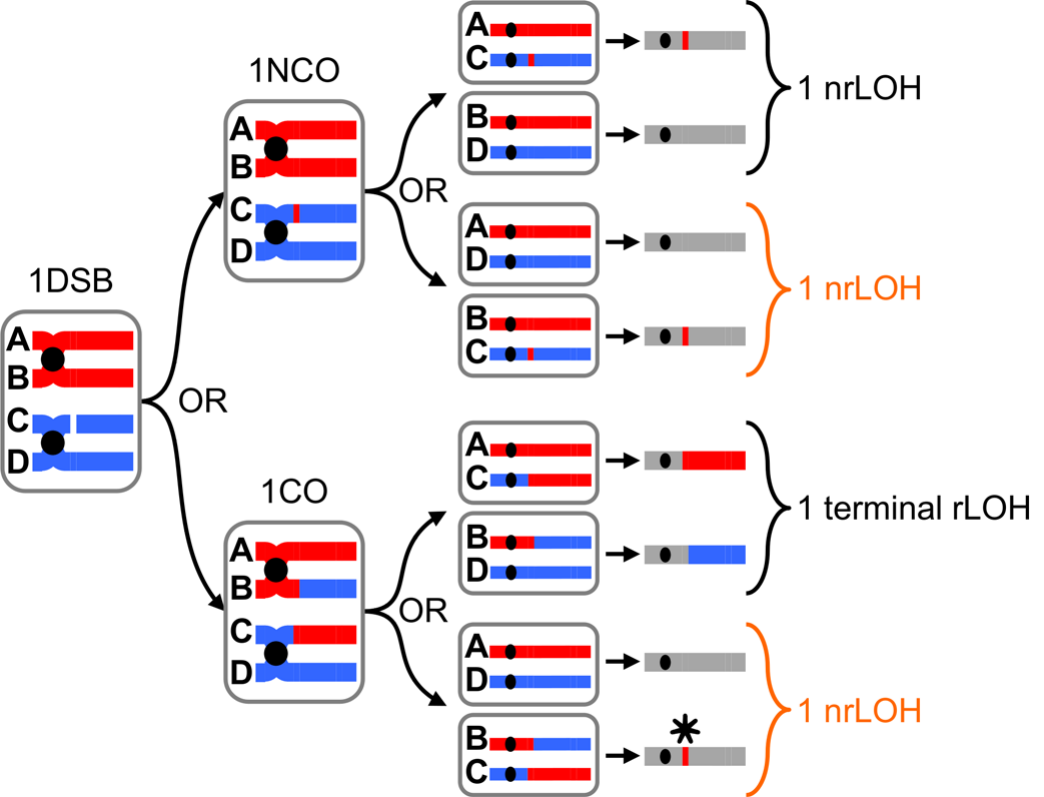
Outcome of recombination events in diploid RTG cells. After DNA replication, each homolog is composed of two identical sister chromatids [A and B for the S288c chromosome, C and D for the SK1 chromosome] linked in the centromere region (black circle). The meiotic DSB(s) is repaired on one of the non-sister chromatid either by a NCO or by a CO associated (or not) with a GC. Depending on the relative segregation of the chromatids in the RTG cells, different outcomes are expected: a non-reciprocal LOH (nrLOH) or a reciprocal LOH (rLOH). The genotypic outcomes when a single DSB is formed on chromatid C and repaired either as a NCO or a CO (+GC) using the chromatid B are illustrated. Asterisk: the CO is masked by the chromosome segregation of the reciprocal products in the same cell while the GC associated with this CO leads to a nrLOH that is undistinguishable from a NCO-induced nrLOH.

The acquisition of the genome-wide recombination profile of the RTG-M and–D pairs provides unprecedented information on the nature of the recombination events (gene conversions and/or crossovers). Due to the occurrence of a single equational division that occurs when the cells exit from the prophase-I of meiosis resulting in RTG diploid cells, the method to detect the gene conversion and crossovers by genotyping is different than in the four haploid spores derived from a meiotic tetrad [2,19,28,33,35–39]. The expected outcome of a single meiotic DSB repair by gene conversion and/or a crossover in a RTG pair is illustrated in Fig 5. DSB repair event by gene conversion is detected by a 3:1 segregation pattern of the SNP positions in the pair of RTG strains and is manifested by a non-reciprocal LOH (nrLOH). Differently, a crossover is detected by the occurrence of reciprocal tracts of LOH (rLOH) in the RTG pair, where the SNP positions segregation pattern is 2:2. The bioinformatics pipeline developed to detect gene conversions and/or crossovers events in diploid strains is shown in S9 Fig.

To estimate the number of crossovers per RTG, we analyzed the SNP positions segregation pattern in the 15 mother and daughter RTG pairs. The tracts of homozygous SNP positions that define the rLOH regions are comprised of two subclasses illustrated in Figs 3B and 5: (i) the terminal rLOH (trLOH), in which one end likely corresponds to the crossover site and the homozygosity extends to the end of the chromosomal arm (formally to the ultimate SNP position), and (ii) interstitial rLOH (irLOH), where both ends of the homozygous tract are flanked by heterozygous tracts, thus reflecting the occurrence of two consecutive crossovers on the same chromosomal arm. The double crossover can involve 2, 3 or 4 chromatids, which is not distinguishable in diploid genotyping. Altogether, we observed 70 trLOH and 66 irLOH (S6 Table). Assuming that each trLOH reflects the occurrence of one crossover and each irLOH reflects two crossovers, we detected a total of 202 COs in total, ranging from 1 to 54 COs per mother-daughter pair.

Concerning the frequencies of gene conversion events (GC), we found that 1.4% of the SNP positions exhibited a 3:1 segregation pattern, leading to non-reciprocal tracts of LOH (nrLOH) as illustrated for the RTG11-M and -D strains in Fig 3. Altogether, among the 15 RTG mother-daughter pairs, we identified a total of 913 nrLOH tracts (mean length of 2.3 kb), varying from 5 to 139 events per pair (S8 Fig and S7 Table). Once again, the nrLOH tracts can be interstitial or terminal. Not surprisingly, the vast majority of the nrLOH is interstitial (847/913 = 93%), and corresponds to gene conversions, the canonical product of meiotic DSB repair by homologous recombination. We observed that 164 interstitial nrLOH were located at the boundary of rLOH events, reflecting crossovers associated with gene conversions, while the remaining 683 are independent of rLOH events, reflecting NCOs. The terminal nrLOH events (66/913) may be true terminal nrLOH events or may be interstitial if they ends in the non-genotyped repeated sub-telomeric regions of the chromosomes. These terminal nrLOH events may result from Break-Induced replication (termed terminal NCO or terminal gene conversion [2,40,41]). Thus, among the 15 RTG pairs, we detected a total of 951 recombination events: 202 COs, including 164 COs associated with GC (81%) and 38 COs not associated with a GC (19%), and 749 NCOs (GC not associated with a detectable CO).

### Masked crossovers further contribute to the diversity of the RTG haplotypes

Due to the random segregation of the non-sister chromatids during the equational RTG division, additional COs may remain undetected upon SNP positions genotyping. As illustrated in Fig 5, upon equational segregation, a single CO leads to rLOH distal to the CO site in only half of the cases in mitotically growing cells, and therefore remains undetected in half of the cases [10,42], while a GC leads to nrLOH regardless of the chromatid segregation. Consistently, all NCOs will be detected as independent nrLOH, while, according to the chromatid segregation, half of the GC associated with a CO (81% of observed COs) will be detected as such (nrLOH at a boundary of a rLOH, i.e a GC associated with a CO), and half will be detected as an independent nrLOH (NCO, or GC not associated with a detectable CO). However, as illustrated in S10 Fig, the probability of CO detection is dependent on the number of CO per chromosome arm; It gradually increases from ½ to ⅔ when more COs occur on the same chromosomal arm. Hence, assuming a random chromatid segregation pattern, and depending on the distribution of CO per chromosome arm, we expect that between ½ and ⅓ of the COs should remain undetected because they do not manifest as a rLOH event. As well, the number of COs will also affect their distribution leading to interstitial or terminal LOH; as the number of COs increases, the probability of interstitial rLOH increases compared to that of terminal rLOH (S10 Fig). Taking into account these parameters, we estimate that the real number of CO in all 15 pairs ranges between 404 (202÷½) and 303 (202÷⅔). Since approximately 81% of the observed COs are associated with a GC, the corrected number of CO associated with a GC might range between 327 (404x0.81) and 245 (303x0.81), and therefore the number of NCO ranges between 586 (913–327) and 668 (913–245). Altogether, this leads to an excess of NCOs over COs of 1.45-fold (327/404) to 2.21 fold (245/303), a ratio opposite to the outcome of uninterrupted meiosis (see Discussion).

To confirm the existence of these masked COs, we induced the sporulation of four RTG pairs (RTG7M-D, RTG8M-D, RTG9M-D, RTG10M-D) showing various extent of recombination frequencies (S8 Fig) and sequenced all four spores arising from one tetrad each. As an example, the genotype of the RTG10-M and RTG10-D pair is illustrated in S11A and S11B Fig and the corresponding tetrads in S11C and S11D Fig. The SNP positions exhibited an expected Mendelian segregation pattern: the homozygous SNP positions of the RTG parental strain segregate 4:0 in the corresponding tetrad (99.69%), and the heterozygous SNP positions exhibit a 2:2 –or, occasionally, a 3:1 –segregation pattern (99.72%), validating our bioinformatics pipeline of diploid cell genotyping. As anticipated, we identified several masked crossovers present in the parent RTG that are revealed in the RTG tetrad by the presence of 2 pairs of reciprocal recombinant molecules among the four spores of the tetrad (S11C and S11D Fig). Altogether, this revealed 37 masked COs (ranging from 0 to 16 per RTG strain) that did not lead to rLOH (S11E Fig), in addition to the 77 COs leading to rLOH, which corresponds to the observed detection frequency of 77/(37+77) = 67.5%, not significantly different from the expected ratio of 60.5% calculated from the distribution of CO per chromosome arm detected in those 4 RTG pairs (p-value = 0.22, Fisher exact test, S8 Table). Two other observations should be mentioned. First, we observed that 86% (32/37) of the masked COs are associated with an adjacent GC. Association with GC is not statistically different between the detected (81%) and masked COs (86%) (p-value = 0.75, Fisher exact test). Second, we observed that in each RTG pair, the number of masked COs is quantitatively related to the number of COs readily identified; namely, in RTG7M-D, RTG8M-D, RTG9M-D, RTG10M-D respectively, we detected 54, 8, 3 and 12 COs by LOH analysis and, we identified 27, 2, 1 and 7 masked COs upon tetrad sequencing. Altogether, these results suggest that the detected and masked COs do not mechanistically differ but simply reflect the way the sister chromatids mitotically segregate upon RTG.

### Iteration of the RTG process increases genome homozygosity

In the absence of recombination between the centromere and the mating type locus on chromosome III, the RTG strains remain heterozygous *MAT***a**/*MAT*α, making it possible to repeat the RTG process. Indeed, by phenotypic analysis of the mating behavior of the RTG strains, coupled with bioinformatic analyses, we found that 32/36 RTG strains were *MAT***a**/*MAT*α, while the others were homozygous at the *MAT* locus, as *MAT***a**/*MAT***a** (RTG11-M and RTG15- M) or *MAT*α/*MAT*α (RTG11-D and RTG15-D) (S2 Table). Consistently, the *MAT* heterozygous (*MAT***a**/*MAT*α) RTG strains sporulated while the *MAT* homozygous strains did not. The rarity of exchanges between the centromere and the *MAT* locus on chromosome III is consistent with the unusually low frequency of meiotic DSB formation in this ~100kb region [15,43].

**Fig 6:**
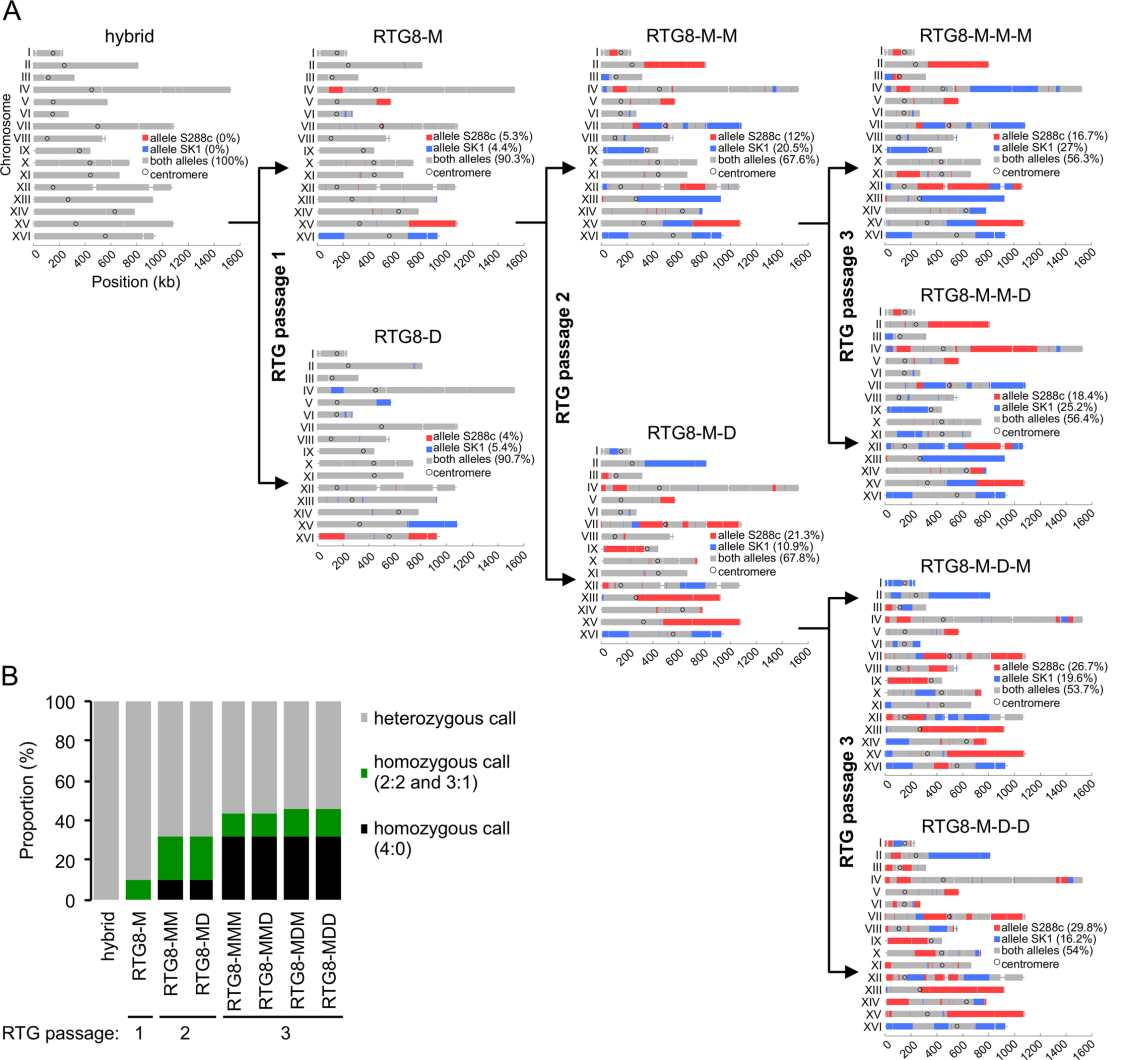
Genotype of RTG cells generated upon iteration of the RTG process. (A) At each passage, the parental strain was subjected to RTG and the mother-daughter RTG cells separated by micromanipulation as described in Materials and Methods and Fig 1. Thus, the parental hybrid strain (AND1702) gave rise to the RTG8-M and RTG8-D cells at passage 1. Similarly the RTG8-M strain gave rise to the RTG8-M-M and RTG8-M-D cells at passage 2. Finally, the RTG8-M-M strain gave rise to RTG8-M-M-M and RTG8-M-M-D at passage 3. The same protocol and nomenclature were applied for the other strains of this lineage. (B) Evolution of the SNP position status along the lineage. At each passage, the pre-existing LOH regions (homozygous SNP positions in a given strain) are transmitted to both RTG descendants and novel reciprocal LOH are generated. Thus, the RTG iteration increases the proportion of LOH regions in the genome, drifting towards one or the other genotype (S288c in red or SK1 in blue).

To examine the genome dynamics of the RTG strains throughout successive passages of RTG, we conducted a RTG pedigree analysis starting from the RTG8-M strain and induced two additional rounds of RTG events, using the micromanipulation method and determined the cell genotype by WGS. The genotype of the 10 RTG strains generated in this lineage, which all remained diploid, is shown in Fig 6 and S9 Table. As expected, the LOH regions acquired at passage *n* were present in passage *n+1* but additional LOH events appeared, indicating that these recombinant RTG strains retained the capacity to recombine and faithfully perform the RTG process. Remarkably, as shown in one example in the lineage RTG8-M>RTG8-MD>RTG8-MDD, the genome homozygosity levels increased from 9.7% of the SNP positions (36 LOH tracts, including 6 reciprocal ones) in passage 1 to 32.2% of the SNP positions (81 LOH tracts, including 17 reciprocal ones) in passage 2, finally reaching 46% of the SNP positions (117 LOH tracts, including 21 reciprocal ones) in passage 3.

In conclusion, the reiteration of the RTG protocol perpetuates the newly acquired LOH regions in a clonal manner, increases the degree of homozygosity and expands haplotype combinations in an incremental manner from one passage to the other. Overall, extensive mosaic genomes of either parental origin are generated, a feature that raises the question of the potential roles of the RTG process in yeast genome evolution.

### Phenotypic diversification of the RTG strains

The genomic diversity of the recombinant RTG yeast cells has the potential to translate into phenotypic variations. The SK1 parent is prototrophic for leucine and methionine, but auxotrophic for histidine while the S288c strain is auxotrophic for all three traits. By complementation, the hybrid is prototrophic for all three amino acids. We examined these phenotypes among the 36 RTG strains in comparison with the parental strains by scoring their growth on histidine, leucine and methionine depleted media (Materials and Methods). As expected, according to the segregation of the *HIS3*, *HIS4*, *LEU2* and *MET15* alleles, the RTG strains exhibited growth or no growth on the appropriate media (13 His^-^/23 His^+^, 3 Leu^-^ /33 Leu^+^ and 7 Met^-^/29 Met^+^) (S10 Table).

**Fig 7:**
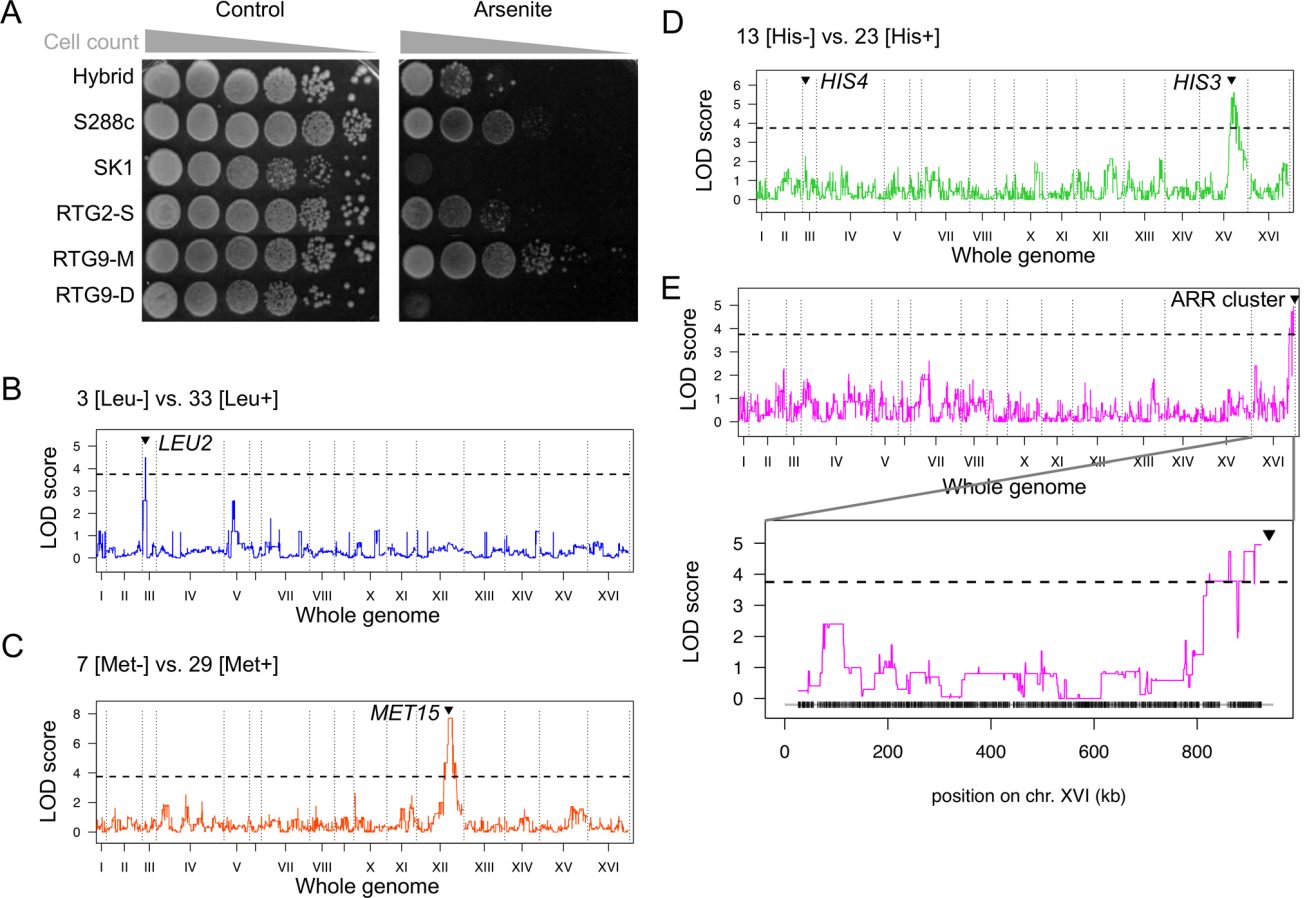
Phenotypic analyses and trait mapping using 36 RTG diploid strains. (A) Semi-quantitative measure of growth rate by spot assay (Materials and Methods) for the parental S288c and SK1 haploids, the hybrid S288c/SK1 diploid and the RTG2-S, RTG9-M and RTG9-D diploid cells which are representative of the degree of growth variation observed among the 36 RTG strains. Control: standard medium; Arsenite: standard medium + NaAsO2 1.5mM. (B) LOD (logarithm of odds) score values along the 16 chromosomes for leucine auxotrophy, a monogenic binary trait. A region of 10kb [chr. III: ~65-76kb] shows a significant association with leucine auxotrophy and includes the expected causal *HIS4-LEU2* locus [chr. III: ~66-68kb]. (C) LOD score values along the 16 chromosomes for methionine auxotrophy, a monogenic binary trait. A region of 265kb [chr. XII: ~627-892kb] shows a significant association with methionine auxotrophy and includes the causal *MET15* locus [chr. XII: ~733-734kb]. (D) LOD score values along the 16 chromosomes for histidine auxotrophy, a digenic binary trait. A region of 219kb [chr. XV: ~690-909kb] shows a significant association with histidine auxotrophy and includes one causal locus *HIS3* [chr. XV: ~722 -723kb]. However, no significant association is found with the second causal locus *HIS4-LEU2* [chr. III: ~66-68kb]. (E) LOD score values along the 16 chromosomes for arsenite resistance. Coordinates of the unique significant linkage interval: chr. XVI: ~819–925 kb. The zoom-in outlines the LOD peak value towards the end of chromosome XVI. The ARR (ARsenicals Resistance) cluster (black arrow head, coordinates chr. XVI: ~938-941kb) is a cluster of three genes, *ARR1*, *ARR2* and *ARR3*, located distal to our ultimate genotyped SNP position of this chromosomal arm (chr. XVI: 928,226bp). The ARR cluster of genes is present in the S288c strain background but absent in SK1.

Additionally, to assay complex multi-factorial traits [44,45], we examined the phenotype of the RTG strains with respect to arsenite resistance using the spot dilution assay (Materials and Methods) (S10 Table). We observed that the SK1 parent is highly sensitive to 1.5mM NaAsO_2_ while the S288c parent is resistant. The hybrid strain shows an intermediate resistance between S288c and SK1. Remarkably, the 36 RTG strains exhibit variation in the strength resistance to arsenite, which we scored in five phenotypic categories (Fig 7 and S10 Table). Certain RTG strains (RTG2-S and RTG9-D) resemble the parental haploid strains while others exhibit increased resistance as compared to the parents (RTG9-M).

### Quantitative trait mapping using the recombinant RTG strains

To map the causal locus, for each auxotrophic trait, the genotype/phenotype relationship was assayed at each SNP position by linkage analysis (Materials and Methods). For each trait, a single significant linkage interval, overlapping in each case the known causal locus (a region of ~10kb overlapping *LEU2*, a region of 265kb overlapping *MET15* and a region of 219 kb overlapping *HIS3*, respectively), was mapped (Fig 7B–7D). Surprisingly, for the digenic histidine auxotrophy phenotype, a genetic linkage at the *HIS4* locus was not observed. Examination of the individual RTG genotype/phenotype relationship led us to observed that although RTG20-D is homozygous for *his4Δ*::*LEU2* allele, and therefore histidine auxotroph, it carries heterozygous genotype at SNP position in the vicinity of *HIS4* locus, suggesting that RTG20-D histidine auxotrophy resulted from a NCO event involving the *HIS4* locus. Thus, we investigated if exclusion of the RTG20-D strain from the analysis improved the mapping. It did not, rather suggesting that the small size of our population does not give enough power to map this second locus. With respect to the segregation of a polygenic trait as arsenite resistance, the statistical association between genotype and phenotype allowed to map a significant QTL with an interval size of 106kb, which span the subtelomere of chromosome XVI (Fig 7E). Consistently, this region includes the well characterized *ARR* (ARsenicals Resistance) gene cluster, a major QTL known to control arsenate resistance [45,46]. The ARR cluster of genes is polymorphic in various strain backgrounds, herein present in the S288c strain background but absent in SK1 [45]. Altogether, these phenotyping and genotyping results provide a proof of concept that quantitative trait mapping in diploid strains can be performed using RTG strains, even with a small set of sequenced samples.

## Discussion

Various aspects of budding yeast “Return To Growth” have been previously studied [11–14,16–18] but the singularity of this process remains to be better understood both at the physiological and molecular levels. Here, we used whole genome sequencing and single cell micromanipulation to comprehensively examine the genome-wide recombination dynamics of RTG hybrid cells. Physiologically, the cells induced to enter meiosis upon carbon and nitrogen starvation, replicate their genome and experience meiotic DSB formation in the prophase-I of meiosis. But when withdrawn before the MI reductional division, the cells rapidly disassemble the synaptonemal complex [18], repair the DSBs [17,18], degrade most meiotic transcripts [47], return to a G1 pattern of gene expression [47], switch to a modified mitotic cell cycle to bud without re-replication [16,17] and finally equationally segregate the (recombined or not) sister chromatids [16,17] into two separated diploid descendants. Precise single cell micromanipulation of the mother and first bud/daughter cells led us to analyze the genotype of the four chromatids that exit from the prophase-I of a single meiosis, before the meiotic cell could become irreversibly engaged to complete meiosis and form haploid spores. We found that each meiotically induced S288c/SK1 *S*. *cerevisiae* diploid cell generates two genetically distinct RTG diploids with mirrored recombinant genotypes. Remarkably, recombination is extensive; the number and the position of the recombination events that give rise to LOH regions are highly variable from one RTG cell to another, while additional “masked” crossing overs events lead to heterozygous yet recombinant genotypes that further increase the haplotype diversity of the individual RTG cells. Thus, at a population level, the process of RTG is able to achieve a massive genetic diversification of diploid yeast cells that is rare in mitotically growing cells and is usually limited to a single chromosomal arm per cell [40,41].

### Insights into the mechanisms of RTG recombination

Four observations demonstrate that RTG recombination is initiated by the numerous Spo11 DSBs that form in the meiotic mother cell: (i) the absence of RTG recombination in the *spo11* mutant [48], (ii) the multiplicity of the recombination events involving several chromosomes ([13], this work), (iii) the multiplicity of exchanges on the same chromosomes leading to a large variety of mosaic haplotypes (this work) and (iv) the frequent recovery of inter-homolog recombination ([13], this work), a hallmark of meiotic recombination that creates the genetic diversity of the gametes. However, how the unrepaired meiotic DSBs and/or the meiotically engaged recombination intermediates are repaired during RTG is still poorly understood. Limited mutant analyses have been reported. Concerning the early steps of DSB processing and strand invasion, the study of Zenvirth et al. [18] showed that the *rad50S* mutant cells, which accumulate unresected DSBs with covalent attachment of Spo11, sharply lose viability upon RTG, supporting the conclusion that these unprocessed DSBs are not repaired in RTG. Similarly, the *rad51* cells, which accumulate resected DSBs, also lose viability in RTG and give very few recombinants [18]. In contrast, the *dmc1* cells, which accumulate hyper-resected DSBs, do not lose viability in RTG and exhibit only slightly reduced recombination levels, up to 30–50% of wild type levels [18]. The fate of the subsequent intermediates, further engaged in the recombination process but not yet resolved, has been examined using the benefit of the *ndt80Δ* mutation, that allows the accumulation of Joint Molecules (JMs) [17,49]. Clearly, these cells retain viability after RTG and efficiently repair the DSBs but their resolution yields reduced CO and increased NCO formation. The study of Dayani et al. [17] revealed the prominent role of the Sgs1-dependent pathway during RTG that processes the JMs by dissolution and produces only NCOs, and the limited formation of COs depends on the Mus81/Mms4 structure selective endonucleases that resolve the JMs into NCOs and COs in an unbiased way. Many other DSB repair intermediates are known to form during meiotic DSB repair [50] but how they are processed during RTG remains to be studied.

Here, in a wild type strain, the genotyping of 15 pairs of RTGs and the sequencing of their sporulation products allowed us to comprehensively examine the genome wide frequencies and the nature of the RTG recombination events, namely gene conversions and crossovers. Altogether, after correction for undetectable events, among the 15 pairs of RTG-M and RTG–D, we estimated between 327 and 245 COs associated with an adjacent GC, between 77 and 58 COs without adjacent GC, and between 586 and 668 NCOs. In contrast, the rate of mitotic recombination events per cell is much lower, in the order of 10^−6^ per division [41,51–53]. This is also in contrast with the outcome of meiosis characterized in four spore tetrads. In the same hybrid background, Martini et al. [19], characterized 7 tetrads and observed a total of 189 NCO and 511 COs, corresponding to a NCO/CO ratio of 0.37. This is the opposite of the 1.45–2.21 fold excess of NCO versus CO observed in the present RTG cells. This genome wide deficit of COs observed in our RTGs compared to meiotic tetrads is consistent with the genetic and physical analyses at the *HIS4-LEU2* recombination hotspot during RTG compared to meiosis [17]. Moreover, in seven tetrads of S288c/SK1 hybrid, approximately 100 recombination events are detected per tetrad, with limited variation (1.3 fold, from 86 to 116) from one tetrad to another [19]. In contrast, as seen in the RTG pairs, the total number of events varies 30-fold (5 in RTG13-M/-D to 151 in RTG7-M/-D) and affect both the absolute number of NCOs and COs.

Finally, to examine whether the cell-to-cell variation is stochastic or coordinated in the individual RTG cells, we compared the observed number of NCOs and COs for the 15 individual RTG pairs. Clearly, the number of NCOs and COs per cell is correlated (correlation coefficient: R^2^ = 0.69) (S12A Fig). Several non-exclusive hypotheses can explain the variation in recombination frequencies and the nature of the outcome per RTG cell. First, it may originate from the asynchronous formation of the ~160–200 DSBs per meiotic cell that occurs in *S*. *cerevisiae* and evolutionary distant yeast strains [54]. We still do not know whether all DSBs are simultaneously formed during the prophase-I of the individual cells but, if not, when nutrient are added to the population of meiotic cells, it would not be surprising that cells with few or numerous DSBs end up with few or numerous recombination events, respectively. So, perhaps, the RTG procedure is able to capture the asynchrony of DSB formation in wild type cells. We can also hypothesize that the RTG regimen disrupts the initial control of DSB formation or the feedback control dependent on Tel1 [55]. However, there are evidences that DSBs do not form once the cells are transferred to rich medium [17,18].

Alternatively, we also envisage that at the time of RTG induction, cells carry broken chromatids that are at different stages of repair and thus can yield distinct outcomes, controlled on a stage-specific or site-specific basis. Likely, a key decisive parameter is the extent to which the DSBs are engaged in the alternative recombination pathways, in particular whether or not they are irreversibly engaged to use the homolog or the sister chromatid as a repair template. Since we retrieved mother/daughter cells at three time points, t = 4, 5 and 8 h, we examined whether the number of recombination events per RTG cell were correlated with the time of withdrawal from sporulation medium. As reported in S12B Fig, we found no correlation; some “early” and “late” RTG cells contain low or high number of recombination events. It should be stressed that although the SK1 strain is best chosen for its sporulation efficiency and synchrony for meiotic recombination studies, the synchrony of the S288c/SK1 hybrid is still not optimized sufficiently in order to conclude on the above time-related questions at the single cell level. In conclusion, although further studies will be required to address the mechanisms and genetics factors involved in RTG recombination, all previous and present data clearly reveal a wider flexibility in the final recombination outcome in individual RTG cells compared to what is observed in 4-spore meiotic products. Likely, the metabolic context of the RTG cells transiently mixes meiotic and mitotic features, once also referred to as “Meiototic” recombination [56].

### The production of recombinant RTG cells provides an alternative approach for complex trait analyses

Although the capacity of *S*. *cerevisiae* cells to perform Return To Growth after meiotic induction was discovered more than half a century ago, perhaps the usefulness of generating recombinant diploids has not been fully perceived so far. Here, we tested whether RTG cells from a polymorphic hybrid diploid (S288c/SK1) could be used to produce phenotypic variation and to map the causal trait loci. In budding yeasts, several strategies have been developed to characterize complex traits and map QTLs [57–64]. A common strategy is to take advantage of one or several naturally polymorphic strains, build hybrids and analyze a large population of meiotic progeny from single or multigenerational crosses. However, a recurrent problem is that hybrid strains often exhibit low sporulation efficiencies and poor spore viability, to various degrees. The reduced spore viability may be attributed to sequence divergence, and/or structural variations, that affect meiotic recombination and chromosome segregation, as well as to genetic incompatibilities resulting from genetic mixing. The unprecedented advantage of using recombinant RTG strains is to immediately produce diploid strains and thus bypass the problem of generating a viable haploid progeny. As a proof of concept, we phenotypically screened 36 RTGs for Mendelian auxotrophic phenotypes and arsenite resistance complex trait. In this last case, we observed a large quantitative variation of variant phenotypes (Fig 7) and unambiguously mapped the major QTL with a small and unselected sample of RTG strains. Beyond, the efficiency and technical simplicity of the RTG process suggest that the construction of large libraries of RTG recombinant will be feasible and therefore opens the possibility to explore highly complex traits. One limitation of using natural meiotic recombination is that it is not evenly distributed along the chromosomes but the use of targeted meiotic recombination [5] for finer mapping can be envisaged. The other tremendous advantage of using RTG strains for complex trait studies is that it should be applicable to sterile but meiotic DSB proficient strains for which classical genetic studies assisted by sexual reproduction is impossible. Finally, it should be emphasized that this is based on the natural property of *S*. *cerevisiae* cells simply responding to nutrient variations, and therefore, it is most likely that some other yeast species are also able to enter meiosis and return to mitotic growth.

In conclusion, the process of RTG induces a potentially underappreciated diversification of the cell genotype and phenotype, with valuable application for trait studies. We anticipate that the non-GMO RTG process can also be useful for numerous biotechnological applications, using model yeasts as well as more genetically complex yeasts, isolated from natural habitats or domesticated for industrial purposes. Another open question is whether this simple but powerful mechanism of genome diversification, which provides an alternative to meiosis, is occurring in the wild. The RTG protocol is somewhat reminiscent of the fluctuating environments that yeasts experience during the lifecycle in wild settings [65]. If so, we can envision that the RTG process plays an important role in shaping yeast genome evolution and potentially occurs in other unicellular eukaryotes.

## Materials and Methods

### Yeast strains and media

All *S*. *cerevisiae* strains used in this study are S288c and/or SK1 derivatives. Their genotypes are reported in S1 Table. The haploid S288c (FY1338) and SK1 (DAO20-1) parental strains were kindly provided by G. Simchen (The Hebrew University of Jerusalem). The *arg4-Bgl* and *arg4-RV* markers were introduced at the *ARG4* locus in both S288c and SK1 backgrounds by two-step gene-replacement using the PstI digested pMY232 or pNPS308 plasmids, respectively [66]. The replacement of *ARG4* with the mutant alleles was verified by PCR and Southern blots; Genomic DNA was digested with EcoRV or BglII digestions, and probed with a *DED81* fragment. The ORT7235 (S288c, *arg4-Bgl*) and ORT7237 (SK1, *arg4-RV*) transformants were crossed to obtain the AND1702 hybrid diploid. The isogenic diploid strains AND1747 and AND1769 were obtained by mating-type switching upon introduction of the HO-containing replicative plasmid pGAL-HO in the ORT7235 and ORT7237 strains, galactose induction and mating [67]. All strains were cultivated on standard media [68].

### Phenotypic analyses

The prototrophy/auxotrophy phenotypes of all strains was assayed by standard replica plating on SC-His, SC-Leu and SC-Met media. Growth was scored as 0 (no growth) or 1 (growth). The arsenite resistance phenotype was assayed by drop test as previously described [64] by plating serially diluted cells on YPD (control) and on YPD + NaAsO_2_ 1.5mM. Growth in presence of NaAsO_2_ was scored from 0 (poor growth) to 4 (strong growth).

### Sporulation

Diploid strains were streaked from -80°C stock onto YPD, and single colonies were patched onto YPGlycerol medium to confirm that they were competent for mitochondrial respiratory function. Sporulation was performed as previously described [69]. In brief, cells from a 5 ml saturated YPD culture were diluted into 100 ml SPS at a density of 10^5^ cells/ml and grown to 2–4.10^7^ cells/ml at 30°C with shaking at 250 rpm. The cells were washed and diluted into 200 ml sporulation medium (1% Potassium Acetate and required amino-acid supplements) in a 2 l flask and shaken at 250 rpm at 30°C to induce sporulation.

### Monitoring of meiotic landmarks

Meiotic progression was monitored by fixing 500 μl of cells in 1.25 ml ethanol and staining the nuclei with 0.5 μg/ml 4’,6-diamidino-2-phenylindole (DAPI) for 30 minutes. Fluorescence microscopy was then used to determine the fraction of bi-nucleate (post-MI) and tetra-nucleate (post-MII) cells. After 48 h in sporulation medium, the sporulation efficiency was determined by phase contrast microscopy as the percentage of tetrads in the culture. Spore viability was measured by dissection of four-spore tetrads. The kinetics of recombination was monitored physically and genetically using the *arg4-Bgl* and *arg4-RV* heteroalleles [24]. For the physical assay, meiotic chromosomal DNA was extracted, digested with Eco*RV* and Bgl*II*, and analyzed by Southern blot as described [70], using the Eco*R*V–BglII (1,016 bp) *ARG4* internal fragment as probe. The production of Arg^+^ cells was monitored by the RTG plating assay (see below).

### Isolation of RTG cells

To isolate RTG cells, samples of the sporulation culture were harvested at various time points from 0 to 24 h after transfer into the sporulation media, and RTG cells isolated using two complementary methods illustrated in Fig 1. The first method called “isolation by prototroph selection”, corresponds to the traditional RTG plating assay [11,13] based on the selection of intragenic recombinants after transfer of heteroallelic auxotrophic cells (here *arg4*-RV and *arg4*-*Bgl* heteroalleles) from a sporulation time course to the selective medium (SC-arginine). The meiotic cells were taken at different times, washed and diluted in H_2_O, and plated onto SC-Arg and YPD plates (~10^4^ and ~10^2^ cell/plate, respectively). The plates were incubated 3 days at 30°C. For each time point, the frequency of heteroallelic recombination at the *ARG4* locus was determined by the ratio of Arg^+^ colonies on SC-Arg/colonies on YPD plates. Since the entry into sporulation and the synchrony in the cell population is not absolute, this mode of selection does not distinguish between: (i) fast sporulating cells that passed the commitment point to sporulation and produced recombinant spores, (ii) a recombinant RTG cell that entered the meiotic prophase-I and returned to growth before commitment or, (iii) a mitotic recombination in a cell that did not enter sporulation. Recombinant RTG cells and recombinant spores can be differentiated because RTG cells result from an equational chromosome segregation and therefore are diploid, while spores that completed meiosis are haploid. In the absence of recombination between the *MAT* locus and the centromere (chr. III), mitotic and RTG cells remain heterozygous for *MAT* and therefore can be screened as non-maters while haploid spores are either **a**-mater or α-mater. Thus, the Arg^+^ colonies were screened for non-mater phenotype (indicative of heterozygosity at the *MAT* locus and therefore diploidy). To ensure that the likely-RTG colonies were the product of meiotic but not mitotic recombination, the Arg^+^ colonies were also screened for histidine, leucine or methionine auxotrophy on SC-His, SC-Leu and SC-Met plates, respectively.

In the second method, called “isolation by mother-daughter micromanipulation”, cells harvested at various time points in sporulation were washed in H_2_O and unbudded cells (40–80 per time point) were individually deposited onto YPD plates using a dissecting microscope (Singer MSM system). The plates were incubated at 30°C and regularly observed until bud formation was complete. Then, the mother and daughter cells were separated when a second bud was visible on the mother cell, i.e. between 4h to 7h after deposition of the meiotic cells on the YPD plate. At that stage, the mother cell is rounder, bigger and re-buds first, while the daughter cell is more elongated, smaller and not yet budded, as previously described [16,17]. Then, the mother and daughter cells were incubated 3 days at 30°C to form colonies, and phenotypically analyzed for mating and auxotrophic phenotypes (in this situation the mating type serves as a recombination marker).

### Whole genome sequencing and read mapping

Genomic DNA was prepared from single-colony culture as described [71] and sequenced on the NGS platform of the Institut Curie, using the V4 and 5500 SOLiD (Life Technologies) or HiSeq2500™ (Illumina) instruments following the manufacturer’s standard protocols. Libraries were constructed for paired-end sequencing (50x35 bp, 75x35 bp or 100x100 bp) or for mate-pair sequencing (50x50 bp). Sequencing data were aligned onto the SGD reference genome (R64 from 2011-02-03 on SGD website, or SacCer3 on UCSC genome browser), using Lifescope (v2.5) (Life Technologies) local alignment algorithms for SOLiD data and BWA (v0.6.2) [72] for HiSeq data (with options “aln -n 0.04 -l 22 -k 1 -t 12 -R 10”).

### Sequencing depth coverage and chromosome copy number analysis

PCR duplicates were filtered-out from mapped sequencing reads using MarkDuplicates tool from Picard [http://broadinstitute.github.io/picard/]. The number of read per genomic position was determined using genomeCoverageBed tool from BEDTools [73], and averaged per 10kb window to detect copy number variation along and between chromosomes. The coordinates of copy number variations were determined using the Control-FREEC software [29].

### Determination of the SNP list

SNP calling was made on the mapped sequencing reads from FY1338 and DAO20-1, using the software implemented in the BioScope (v1.3) framework, with, in addition to default parameters, "High" stringency criterion (i.e. calls should be detected on both DNA strands). We obtained 115 calls for FY1338 and 65,134 calls for SK1. The common calls found in both parental strains were filtered out (53 each), as they represent SNPs of the reference SGD strain that do not discriminate the S288c and SK1 strain backgrounds. Then, heterozygous calls and calls with a score higher than 5.10^−7^ were removed (32 for FY1338 and 1,180 for DAO20-1), giving a list of 63,901 polymorphic positions differentiating DAO20-1 from SGD reference genome. This list of polymorphisms was further filtered based on the experimental genotyping results (see below for method) from the sequencing of the hybrid diploid (AND1702) and of two haploid parents of each background (FY1338 and ORT7235 for S288c, DAO20-1 and ORT7237 for SK1). 62,218 SNP positions having the expected genotype (heterozygous, homozygous S288c, or homozygous SK1 respectively) were retained. Among the eliminated SNP positions, 12 SNP positions in the *ARG4* region had the genotype “homozygous S288c” in ORT7237 and in AND1702 (due to the introduction of the *arg4*-*RV* allele in ORT7237), and the 77 mitochondrial SNP positions were homozygous in the hybrid AND1702 strain, which exclusively inherited the mitochondrial DNA from the S288c parental strain.

### SNP position genotyping

All RTG strains were genotyped for the robust 62,218 polymorphic SNP positions defined above. The reads covering the polymorphic positions were selected using intersectBed tool from BEDTools [73]. The position and the identity of the polymorphism(s) covered by each read were computed. The base at the designated position was extracted and compared to the base found in SGD reference genome, and in the list of SK1 polymorphisms. The number of reads carrying the S288c allele or the SK1 allele was recorded. A genotype was attributed only if coverage was greater than 5X and if at least 2/3 of the reads display the parental alleles. Genotyping criteria to determine thresholds (described in S5 Fig) were set up based on the distribution of the allelic frequencies observed in 5 control sequencings (FY1338, ORT7235, DAO20-1, ORT7237 and AND1702 strains). A given position was genotyped “S288c” if >95% of the reads exhibited the S288c allele; It was genotyped “SK1” if >75% of the reads exhibited SK1 allele; It was genotyped “heterozygous” if 25–95% of the reads displayed S288c and 5–75% of the reads displayed SK1 allele. These non-symmetrical thresholds, biased/shifted toward S288c, are justified by the alignment against the SGD (S288c) reference genome. Altogether, with these thresholds, >99.5% of the SNP positions had the expected genotype in each of the 5 control sequencings. In diploid samples, a small bias in S288c/SK1 read ratio would transform a heterozygous position into a homozygous call. Therefore, to increase the confidence in genotyping call in diploid strains, only the genotype switched that affect at least 3 adjacent SNP positions were retained. When tetrads from the RTGs were performed, 99.69% of the SNP positions homozygous in the RTG segregated 4:0 in the corresponding tetrad, confirming the robustness of this threshold.

### LOH analysis

Preliminarily, the LOH analysis was solely based on the SNP positions genotype. Consecutive SNP positions with the same homozygous genotype were grouped into LOH tracts. In the RTG4-S and RTG17-D strains, the SNP positions involved in Copy Number Variation (CNV) were excluded from LOH analysis. Then, in addition to the SNP positions genotyping, the LOH analysis was deepen in the pairs of mother-daughter RTG strains, using custom scripts, to include the segregation information. Only the SNP positions robustly genotyped in both the mother and daughter strains were retained. A SNP position heterozygous in both strains, or homozygous with an opposite genotype in each strain, exhibits a 2:2 segregation pattern. Alternatively, a SNP position that is heterozygous in one strain and homozygous in the other exhibits a 3:1 segregation pattern. The few SNP positions that displayed a 4:0 segregation pattern were excluded from the LOH analysis as they likely result from a pre-meiotic gene conversion event. To assemble the LOH regions and determine their coordinates, in a first step, the SNP positions with a 3:1 segregation pattern were set aside and the SNP positions with a 2:2 segregation pattern were grouped in tracts of the same genotype. The ones of homozygous genotype (SK1 in one strain, S288c in the other one) correspond to reciprocal LOH (rLOH). In a second step, all SNP positions were analyzed together, and grouped in tracts of same genotype/segregation pattern. The tracts made of 3:1 SNP positions were defined as non-reciprocal LOH (nrLOH).

### Recombination analysis

We analyzed the recombination events in the RTG strains based on the position of LOH regions. For the single RTG strains isolated by Arg^+^ selection, the genotype switches define the positions of the recombination events, without distinction between CO and NCO. In contrast, in the mother-daughter RTG pairs, we could identify COs (at the boundaries of reciprocal LOH regions), GC associated with CO (non reciprocal LOH region in-between a heterozygous region and a reciprocal LOH region), and NCO (non reciprocal LOH region inside a heterozygous region or inside a reciprocal LOH region). To validate the recombination events in the RTG pairs, we adapted the CrossOver (v6.3) algorithm from ReCombine (v2.1) [27], a suite of programs initially dedicated to the analysis of tetrad data (4 haploid genotypes). To adapt the format of the dataset, the genotype of each diploid was split into two haplotypes (or chromatids) using the following criteria: at homozygous positions, the two chromatids have the same genotype, while at heterozygous positions, systematically the first chromatid is S288c and the second SK1. Thus, we obtain a tetrad-like dataset where 2 chromatids were deduced from the genotype of the mother RTG strain and the 2 others from the daughter strain. The output of CrossOver program was manually corrected (as some events were attributed to no chromatid). The output data were run into the groupEvents program, kindly provided by J. Fung lab (UCSF), to merge closely spaced events as single ones and refine the classification of the recombination events [28]. Complex events were manually verified and reclassified when necessary.

However, due to the random distribution of the chromatids in the mother and daughter cells, depending on the number of CO on the same chromosome arm, only between ½ and ⅔ of the crossovers are detected. When the two recombinant chromatids resulting from a CO segregate away from each other, the resulting cells both exhibit a LOH. When the two recombinant chromatids resulting from a CO co-segregate in the same cell, the other cell inherits of the two parental chromosomes, thus both cells remain heterozygous, and the CO remains undetected. To verify the existence of these potentially “masked” COs, we sporulated 8 RTG strains (4 RTG pairs) and sequenced one 4-spore tetrad for each. The haplotyping of the two RTG chromosomes by haploidization led to the detection of “masked” CO: CO involving two chromatids which segregate in the same RTG cell do not induce LOH distal to the CO site, but lead to four recombinant chromatid upon sporulation. Thus, these tetrads were analyzed with CrossOver and groupEvents programs [27,28], and the recombination events resulting from the sporulation were manually separated from the recombination resulting from the RTG to identified the masked COs.

### Linkage analysis

The trait linkage analysis was performed as described [74] using the R/qtl package [75]. For each trait separately, the QTLs were identified using the LOD scores (log_10_ of the ratio of the likelihood of the experimental hypothesis to the likelihood of the null hypothesis). The linkage was significant when the LOD score was greater than the 5% tail of the LOD scores obtained by 1000 permutations of the phenotype values.

## Supporting Information

S1 Fig Distribution of the SNP polymorphisms in the S288c/SK1 hybrid. (A) Repartition of the SNP density along the 16 chromosomes. Dotted line: average SNP density (51 SNPs/10kb). (B) Distribution of the genome-wide physical spacing between the SNPs.
(TIF)

S2 Fig Sporulation and spore viability in the S288c, SK1 and hybrid diploids. (A) Sporulation efficiency. The percent sporulation was determined by phase contrast microscopy as the number of asci in the meiotic culture after 48h. 1000 cells were examined. (B) Spore viability observed after dissection of 132 4-spore tetrads per strain. (C) Distribution of viable spores among the dissected 4-spore tetrads in (B).
(TIF)

S3 Fig Meiotic progression and timing of recombination in the S288c, SK1 and hybrid diploids. (A) Meiotic progression (%MI+MII cells) monitored by DAPI staining. For each time point, the percentage of mono-, bi- and tetra-nucleated cells was determined by fluorescent microscopy. Sample size: 200 cells per strain. (A, D-F). Grey shading illustrates the window of time (4h to 8h after meiosis induction) chosen for the RTG experiments. (B) Map of the *ARG4/DED81* hotspot. The *arg4-RV* allele contains a 2-bp deletion ablating the EcoRV restriction site (“RV”, indicated by a vertical red line) at position +258 relative to the *ARG4* open reading frame, and *arg4-Bgl*, a 4-bp insertion by fill-in of a BglII restriction site (“Bgl”, indicated by a vertical blue line) at position +1,274. The chromosome VIII is heteroallelic for the *arg4-Bgl* mutation of *ARG4* gene, disrupting either *BglII* (blue: natural site, grey: disrupted site) or EcoRV site (red: natural site, grey: disrupted site). Green vertical arrows mark DSB sites. Numbered boxes indicate the genes: 17: YHR017*w*, 18: YHR018*c* (*ARG4*), 19: YHR019*c* (*DED81*), 20: YHR020*w*. Arrowheads indicate the direction of transcription. An EcoRV/BglII double digest, probed with *EcoRV–BglII* (1016bp) *ARG4* internal fragment (yellow) detects both parental and recombinant bands, however it detects DSBs on the P2 parental fragment only. DSB1 and DSB2 indicate the location of the *ARG4* and *DED81* DSBs, respectively [1,2]. (C) Physical analysis of meiotic recombination at the *ARG4/DED81* hotspot in the hybrid (AND1702), S288c (AND1747) and SK1 (AND1769) diploids. Genomic DNA was extracted at indicated times (h) after transfer to the sporulation medium, digested with EcoRV and BglII and probed with the *ARG4 EcoRV–BglII ARG4* fragment (1016bp) fragment. P1: *arg4-Bgl* parental fragment; P2: *arg4-RV* parental fragment; R1: *ARG4* recombinant fragment; R2: *arg4-RV*,*Bgl* recombinant fragment; DSB1: *ARG4* DSB on the P2 fragment; DSB2: *DED81* DSB on the P2 fragment. (D) Quantitation of the recombinant and DSBs bands shown in panel C [70]. (E) Left: Gene conversion toward the wild-type allele at one of the *arg4* heteroalleles restores a wild type copy of the *ARG4* gene. Right: Formation of Arg^+^ recombinants by RTG in the hybrid diploid. Cells were plated at the indicated time points onto either SC-Arg or rich medium (YPD) plates and incubated for 3 days at 30°C. The frequency of heteroallelic recombination at *ARG4* was determined by the ratio of Arg^+^ colonies/total viable colonies. Cell viability was calculated by dividing the number of YPD colonies by the number of YPD colonies at time zero. (F) Left: recombination between the centromere and the heteroallelic auxotrophy markers revealing the recessive phenotype, by LOH. Right: Formation of auxotrophs by RTG, assayed by replica plating. Only the hybrid strain contains heterozygous genetic markers that can be used for this assay. (G) Viability of the unbudded S288c/SK1 or SK1/SK1 cells during the meiotic time course, or after growth in YPD or SPS media. Top: number of unbudded cells deposited on the YPD plate. Bud appearance was scored within the 8h following deposition on the YPD plate and colonies formation scored after 3 days at 30°C. Green: mother-daughter cells were separated upon bud formation, and both gave rise to a colony. Orange: mother-daughter cells were separated upon bud formation, but only one of the two gave rise to a colony. Light green: no bud was observed, but a colony was formed, likely resulting from the progression of the meiotic cell into spore formation and germination. Grey: no bud was observed, and no colony was formed.
(TIF)

S4 Fig Bioinformatic pipeline to characterize the LOH and recombination events in the RTG pairs. This pipeline is annotated to analyze the Illumina sequencing. For each step, the bioinformatics tools are indicated. ^(1)^ BWA v0.6.2 [3] (For the SOLiD sequencing, the alignment was performed using Lifescope v2.5.)^(2)^ Picard [http://broadinstitute.github.io/picard/]; ^(3)^ BEDtools [4] ^(4)^ Control-FREEC [5] ^(5)^ CrossOver v6.3 from ReCombine v2.1 [6] ^(6)^ groupEvents, kind gift from J. Fung (UCSF) used in [7].
(TIF)

S5 Fig Genotyping criteria. (A) Example of a SNP call and rules applied to determine the genotype. (B) Determination of the genotyping thresholds based on the experimental data. Y-axis: count of sequencing reads. X-axis: percentage of allele specific reads. S288c alleles (red); SK1 alleles (blue); heterozygotes allleles (grey); Blue x-axis: fraction of SK1 frequency, red x-axis: S288c frequency. Note that the distribution of the S228c allele calls is rarely ambiguous because the alignment of the sequencing reads was performed on the SGD reference genome, which is quasi identical to the S288c genome used in the hybrid strain. In contrast, as expected, the call for the SK1 allele is more dispersed due to the lower efficiency of SK1 polymorphic read alignment on the SGD reference genome. Practically, the selected thresholds correspond to the minima experimentally observed in the distribution of the allelic frequencies in the parental and RTG samples.
(TIF)

S6 Fig Chromosomal coverage of the 36 RTG strains. Small chromosomes often exhibits a slightly higher coverage, therefore the visual interpretation of chromosome copy number from the coverage is facilitated when chromosomes are sorted by size. X-axis: The 16 chromosomes are sorted by increasing chromosome size (I, VI, III, IX, VIII, V, XI, X, XIV, II, XIII, XVI, XII, VII, XV, IV). Y-axis: Sequencing read count, averaged per 1-kb window. Note the terminal deletions/duplications in RTG4-S (chromosomes V and XVI) and RTG17-D (chromosomes III and V).
(PDF)

S7 Fig Ectopic recombination in the RTG4-S cell generated a deletion/duplication. (A) Chromosomal depth coverage of the RTG4-S strain, calculated with a 1kb window. As the cell is diploid, the 1.5 fold change in chromosome V corresponds to a duplication, while the 0.5 fold change on chromosome XVI corresponds to a deletion. (B) Coordinates of the breakpoint for the duplication and deletion in RTG4-S were determined using Control-FREEC software [5], and related to the coordinates of the closest Ty (SGD,[8]). (C) Southern blot analysis of chromosome V. Genomic DNA from the hybrid and RTG4-S strains was extracted in plugs and the chromosomes separated by standard PFGE. Ethidium bromide (EtBr) staining indicates the position of each chromosome on the gel. Chromosome V probe: fragment of the *SCC4* gene (coordinate: chromosome V: 462982–464840). The red asterisk indicates the rearranged chromosome V. Instead of a single chromosome V band, in the parental strain, the RTG4-S DNA exhibits an additional band with the size predicted for the chromosome XVI::V non-reciprocal translocation, depicted in panel (D).(D) Ectopic BIR model explaining the gross chromosomal rearrangement that occurred between the Ty1 elements present on the SK1 chromosomes V and XVI. Blue arrowheads: Ty1 elements (from SK1) involved in the BIR.
(TIF)

S8 Fig Genotype map of the 15 RTG-M and -D pairs. For each chromosome, the RTG-M chromosome is shown on top and the RTG-D chromosome below.
(TIF)

S9 Fig Bioinformatics pipeline to characterize the CO and gene conversion (GC) from the LOH events obtained in a RTG mother-daughter pair. (A) Decision tree allowing the classification of a reciprocal LOH (rLOH) relative to its chromosomal position: a terminal rLOH involves the extremity of one chromosomal arm while an interstitial rLOH is internally located. (B) Decision tree allowing the classification of a non-reciprocal LOH (nrLOH) depending on the relative position of the adjacent rLOH: gene conversions associated with CO are located at the border of the rLOH, while NCO are not contiguous with rLOH.
(TIF)

S10 Fig LOH outcome of CO events per chromosome in diploid RTG cells depends on the number of CO. Legends and color code as in Fig 5. (A) Case of 1 CO per chromosomal arm. The numbers adjacent to the panels indicate the ratio of number of detected CO per chromosomal arm versus the total number of CO per chromosomal arm. For example, 0/1 means no LOH is detected albeit one CO occurred. After random sister chromatid segregation, the CO is detected in half of the cases. (B) Case of 2 COs per chromosomal arm. The COs are counted as one when it yields a terminal LOH or two when it yields an interstitial LOH. Globally, the COs are detected in 62.5% of the cases. (C) Case of 3 COs per chromosomal arm. The COs are detected in 62.5% of the cases. (D) Case of 4 COs per chromosomal arm. The COs are detected in 64.1% of the cases. (E) Summary of CO detection per chromosomal arm for 1–10 COs. The detection rate tends to 2/3.
(TIF)

S11 Fig Detection of masked CO upon genotyping of the tetrads issued from the sporulation of RTG strains. (A-B) Genotype of the RTG10-M and RTG10-D strains, respectively. (C-D) Genotype of 4 spore tetrad issued from the sporulation of the RTG10-M and RTG10-D strains, respectively. The recombination events in the RTG tetrad correspond to the sum of the recombination events that pre-existed in the RTG and the additional recombination events that occurred during the sporulation of the RTG. Note that the regions where the RTG parent was homozygous have the same genotype in the 4 spores. The masked crossovers associated with (black boxes) or without (green box) an adjacent gene conversion segregate as pairs of reciprocal products in the tetrad. Most frequently, the recombination events that occurred during the sporulation of the parent RTG involve only 1 or 2 chromatids. In both tetrads, numerous additional recombination events occurred upon sporulation of the RTG strains, indicating that the RTG retained their capacity to perform meiotic recombination and thus further diversify their genome upon one round of RTG followed by a completed meiosis. (E) Number of masked CO revealed by RTG tetrad sequencing. The number of CO detected by segregation analysis in RTG pairs is given, as well as the total number of CO.
(TIF)

S12 Fig Relationship between COs and NCOs in the 15 RTG pairs. (A) The number of observed NCO per RTG pair correlates with the log_10_ of the number of COs (logarithmic regression). (B) The total number of observed events (CO+NCO) per RTG pair does not correlate with the time of RTG induction (linear regression).
(TIF)

S1 Table Parental strain genotypes. (XLSX)

S2 Table Genotype of the 36 RTG strains generated from the hybrid strain AND1702. (XLSX)

S3 Table Sequencing information. The level of PCR duplicates is the percentage of PCR duplicates among all mapped reads. The read count is the number of mapped reads after PCR duplicate removal. PE: Paired-end sequencing, MP: Mate-pair sequencing, NxN: read lengths.
(XLSX)

S4 Table Allelic distribution in the 36 RTG strains. (XLSX)

S5 Table Summary of all recombination events detected and corrected in the 15 RTG pair strains. "Recombination events corrected after estimation of masked CO", "Corrected CO" column: For RTG7-10, the number represents the CO observed by LOH analysis + CO masked detected by sporulation and tetrad genotyping. For RTG11-21, the numbers represent the CO observed by LOH analysis corrected with both ½ or ⅔ factors). Corrected NCO: the numbers are calculated as (nrLOH – 0.81 x corrected CO).
(XLSX)

S6 Table List of rLOH events. (XLSX)

S7 Table List of nrLOH events. (XLSX)

S8 Table Expected and observed frequency of masked COs in the RTG pairs 7, 8, 9 and 10. The expected CO detection frequency correspond to the average of the detection probability of each CO. For example, for RTG8-M/-D, (6 arms * 1 CO * 50 + 2 arms * 2 COs * 62.5)/10 total CO = 55. The observed CO detection frequency is the ratio between masked CO and total CO.
(XLSX)

S9 Table Genotype of the RTG strains isolated after two and three RTG passages. RTG8-M-M and RTG8-M-D are the mother and daughter RTG cells obtained after one RTG passage of the RTG8-M cell isolated from the first RTG passage of the parental strain AND1702. RTG8-M-M-M and RTG8-M-M-D are the mother and daughter cells obtained after one RTG passage of the RTG8-M-M cell. RTG8-M-D-M and RTG8-M-D-D are the mother and daughter cells obtained after one RTG passage of the RTG8-M-D cell.
(XLSX)

S10 Table Phenotyping scores. For the arsenite resistance phenotype, 0–4 values have been attributed based on serial dilution drop-tests (0: no growth, 4: growth at the maximal dilution). For the binary phenotype (histidine, leucine or methionine prototrophy), 0–1 values have been attributed based on replica plating (0: no growth, 1:growth).
(XLSX)

S1 Text Supporting references. (DOCX)
