# Correction: Extensive Recombination of a Yeast Diploid Hybrid through Meiotic Reversion

**DOI:** 10.1371/journal.pgen.1005953

**Published:** 2016-03-18

**Authors:** The PLOS Genetics Staff

The following information is missing from the Data Availability Statement: The genotype of all the parental and RTG strains have been deposited in Dryad, DOI: 10.5061/dryad.9sh82 (http://dx.doi.org/10.5061/dryad.9sh82). There is one table per sample, indicating the *S*. *cerevisiae* chromosome number, the physical coordinate and the parental allele (S288c or SK1) of the SNP markers, respectively. The publisher apologizes for the error.
