# Supplementary figures and images for: Extensive Recombination of a Yeast Diploid Hybrid through Meiotic Reversion
Source: PLoS Genet. 2016 Feb 1;12(2):e1005781. doi: 10.1371/journal.pgen.1005781 (PMC4734685; doi:10.1371/journal.pgen.1005781)

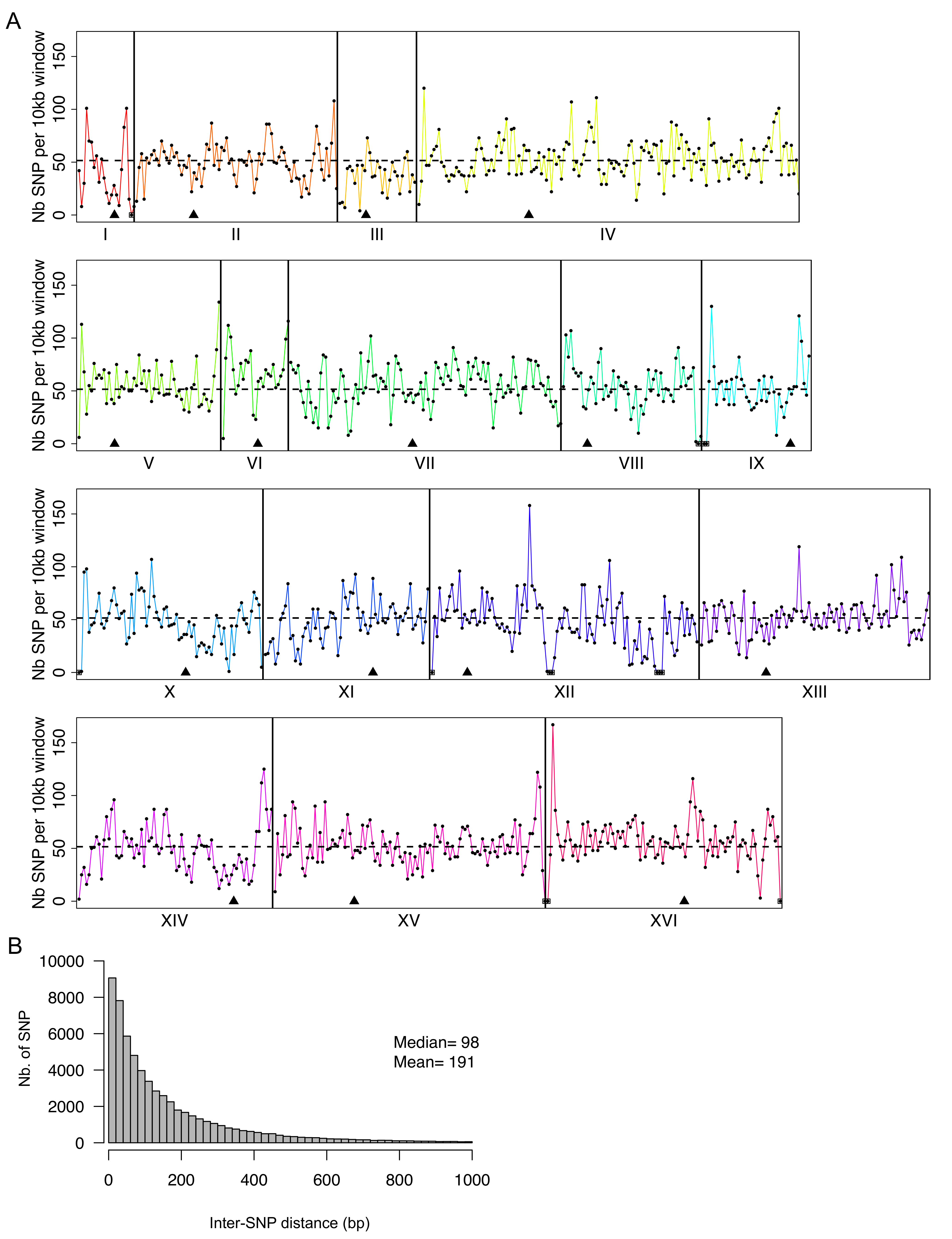

Supplement: S1 Fig — (A) Repartition of the SNP density along the 16 chromosomes. Dotted line: average SNP density (51 SNPs/10kb). (B) Distribution of the genome-wide physical spacing between the SNPs. (TIF) [file pgen.1005781.s001.tif]

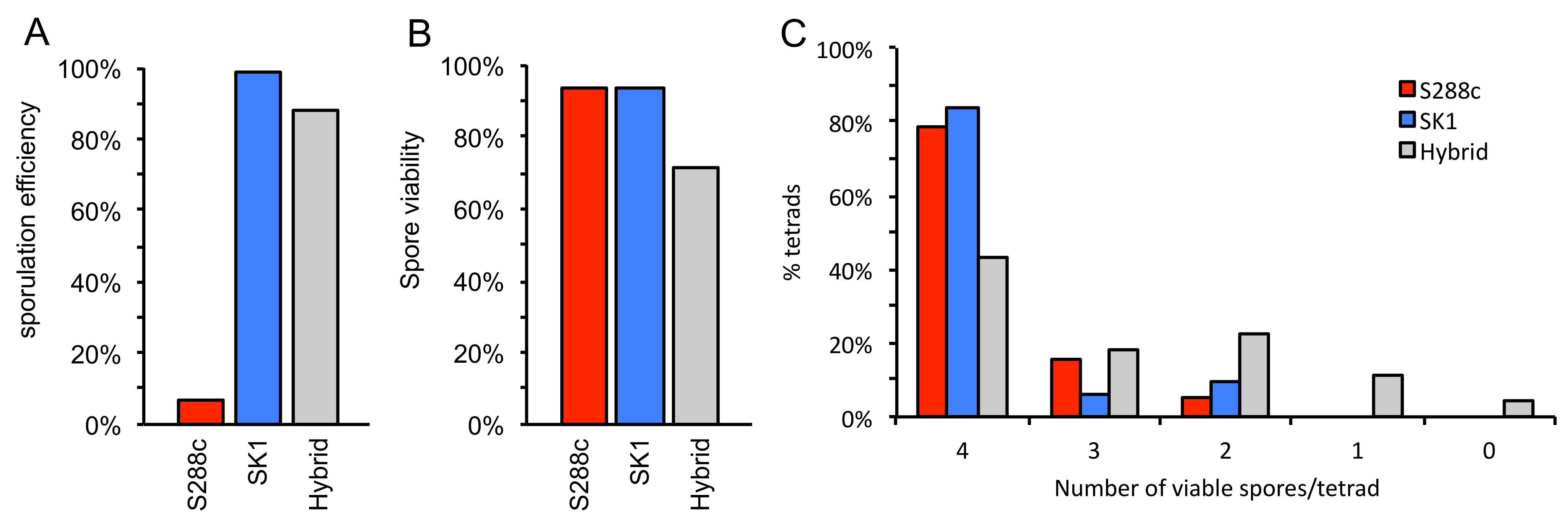

Supplement: S2 Fig — (A) Sporulation efficiency. The percent sporulation was determined by phase contrast microscopy as the number of asci in the meiotic culture after 48h. 1000 cells were examined. (B) Spore viability observed after dissection of 132 4-spore tetrads per strain. (C) Distribution of viable spores among the dissected 4-spore tetrads in (B). (TIF) [file pgen.1005781.s002.tif]

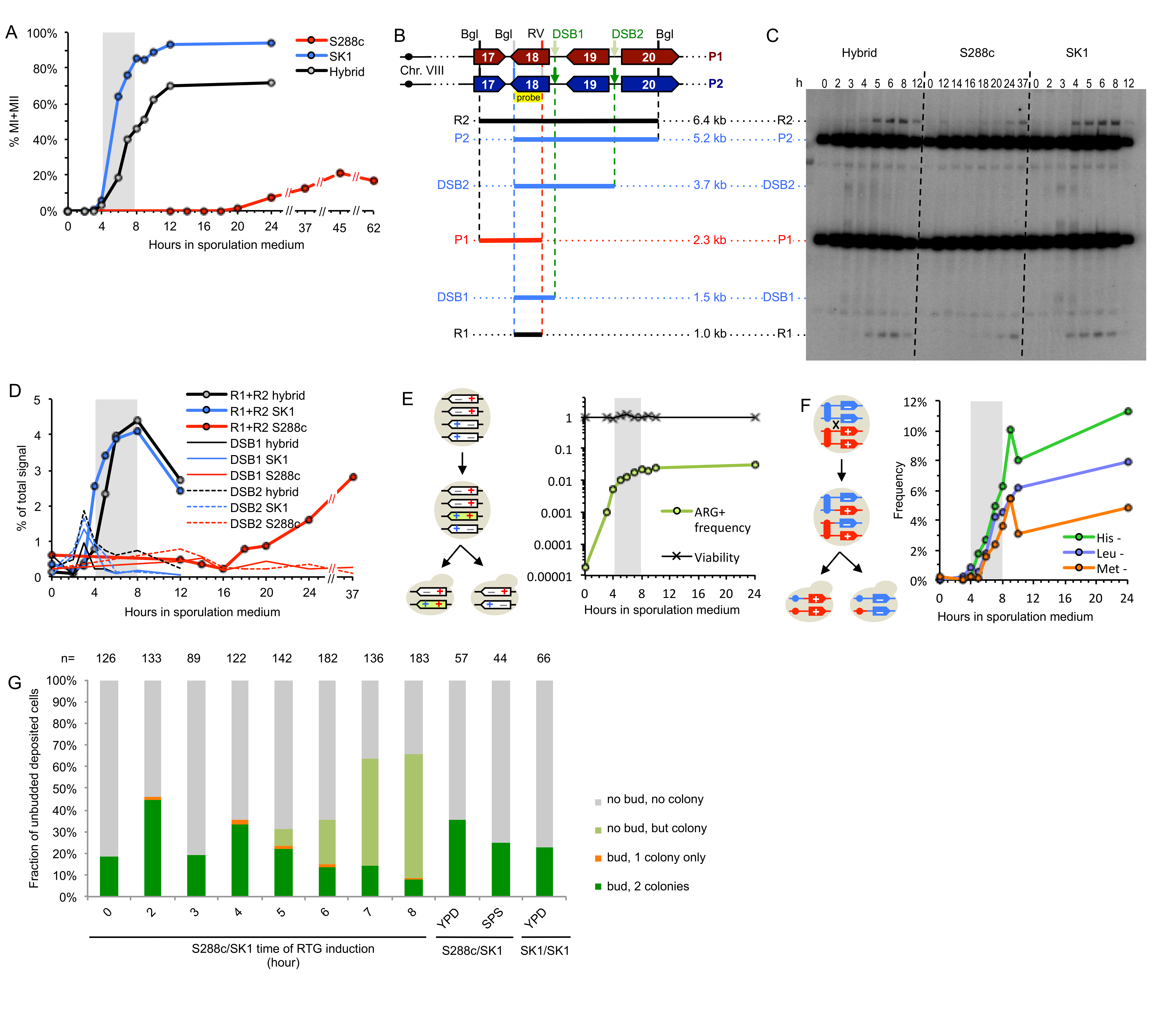

Supplement: S3 Fig — (A) Meiotic progression (%MI+MII cells) monitored by DAPI staining. For each time point, the percentage of mono-, bi- and tetra-nucleated cells was determined by fluorescent microscopy. Sample size: 200 cells per strain. (A, D-F). Grey shading illustrates the window of time (4h to 8h after meiosis induction) chosen for the RTG experiments. (B) Map of the ARG4/DED81 hotspot. The arg4-RV allele contains a 2-bp deletion ablating the EcoRV restriction site (“RV”, indicated by a vertical red line) at position +258 relative to the ARG4 open reading frame, and arg4-Bgl, a 4-bp insertion by fill-in of a BglII restriction site (“Bgl”, indicated by a vertical blue line) at position +1,274. The chromosome VIII is heteroallelic for the arg4-Bgl mutation of ARG4 gene, disrupting either BglII (blue: natural site, grey: disrupted site) or EcoRV site (red: natural site, grey: disrupted site). Green vertical arrows mark DSB sites. Numbered boxes indicate the genes: 17: YHR017w, 18: YHR018c (ARG4), 19: YHR019c (DED81), 20: YHR020w. Arrowheads indicate the direction of transcription. An EcoRV/BglII double digest, probed with EcoRV–BglII (1016bp) ARG4 internal fragment (yellow) detects both parental and recombinant bands, however it detects DSBs on the P2 parental fragment only. DSB1 and DSB2 indicate the location of the ARG4 and DED81 DSBs, respectively [1,2]. (C) Physical analysis of meiotic recombination at the ARG4/DED81 hotspot in the hybrid (AND1702), S288c (AND1747) and SK1 (AND1769) diploids. Genomic DNA was extracted at indicated times (h) after transfer to the sporulation medium, digested with EcoRV and BglII and probed with the ARG4 EcoRV–BglII ARG4 fragment (1016bp) fragment. P1: arg4-Bgl parental fragment; P2: arg4-RV parental fragment; R1: ARG4 recombinant fragment; R2: arg4-RV,Bgl recombinant fragment; DSB1: ARG4 DSB on the P2 fragment; DSB2: DED81 DSB on the P2 fragment. (D) Quantitation of the recombinant and DSBs bands shown in panel C [70]. (E) Left: Gene conversion [file pgen.1005781.s003.tif]

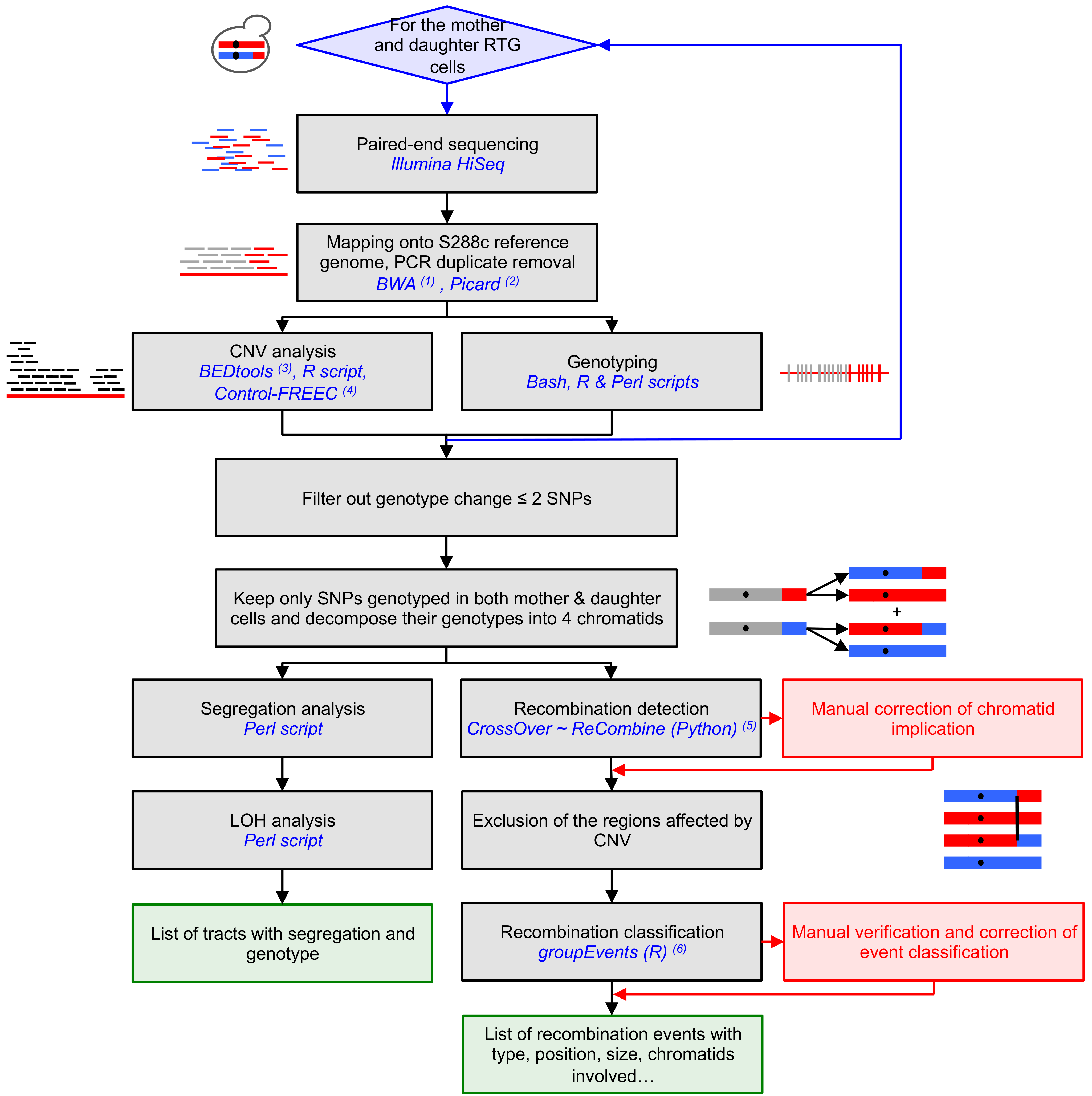

Supplement: S4 Fig — This pipeline is annotated to analyze the Illumina sequencing. For each step, the bioinformatics tools are indicated. (1) BWA v0.6.2 [3] (For the SOLiD sequencing, the alignment was performed using Lifescope v2.5.)(2) Picard [http://broadinstitute.github.io/picard/]; (3) BEDtools [4] (4) Control-FREEC [5] (5) CrossOver v6.3 from ReCombine v2.1 [6] (6) groupEvents, kind gift from J. Fung (UCSF) used in [7]. (TIF) [file pgen.1005781.s004.tif]

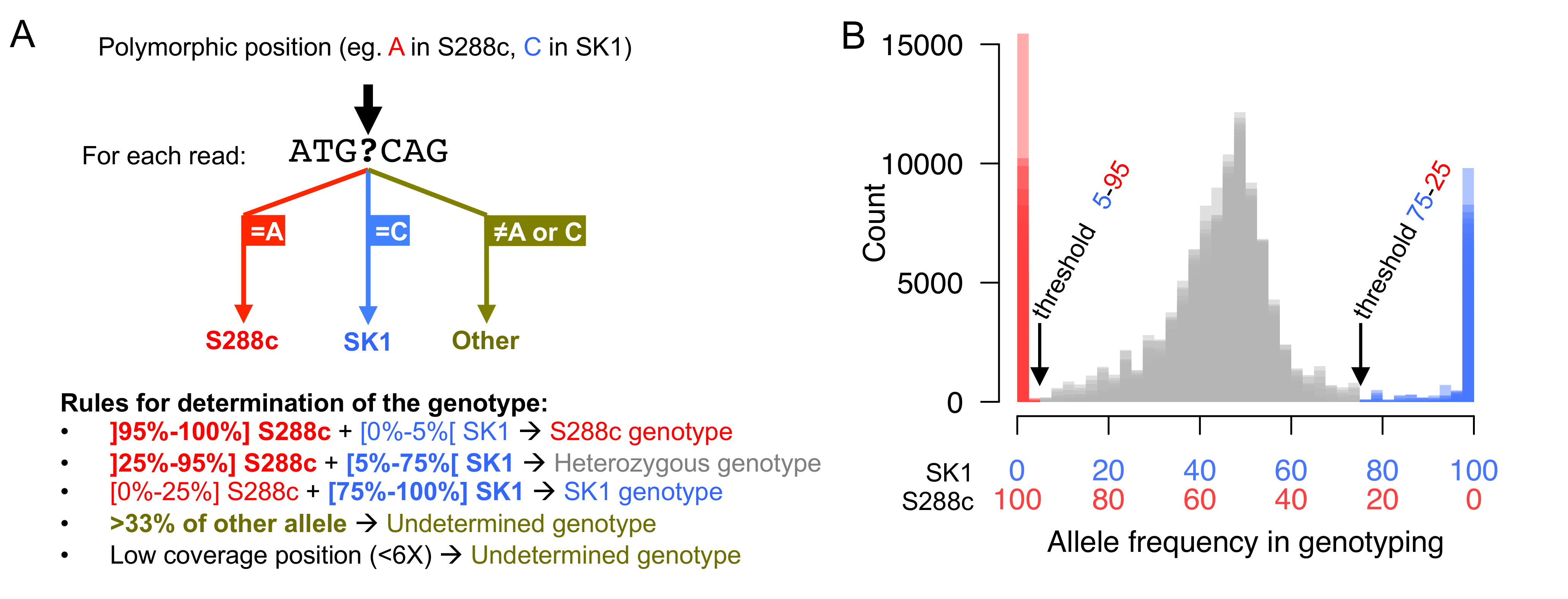

Supplement: S5 Fig — (A) Example of a SNP call and rules applied to determine the genotype. (B) Determination of the genotyping thresholds based on the experimental data. Y-axis: count of sequencing reads. X-axis: percentage of allele specific reads. S288c alleles (red); SK1 alleles (blue); heterozygotes allleles (grey); Blue x-axis: fraction of SK1 frequency, red x-axis: S288c frequency. Note that the distribution of the S228c allele calls is rarely ambiguous because the alignment of the sequencing reads was performed on the SGD reference genome, which is quasi identical to the S288c genome used in the hybrid strain. In contrast, as expected, the call for the SK1 allele is more dispersed due to the lower efficiency of SK1 polymorphic read alignment on the SGD reference genome. Practically, the selected thresholds correspond to the minima experimentally observed in the distribution of the allelic frequencies in the parental and RTG samples. (TIF) [file pgen.1005781.s005.tif]

S6 Fig. part 1

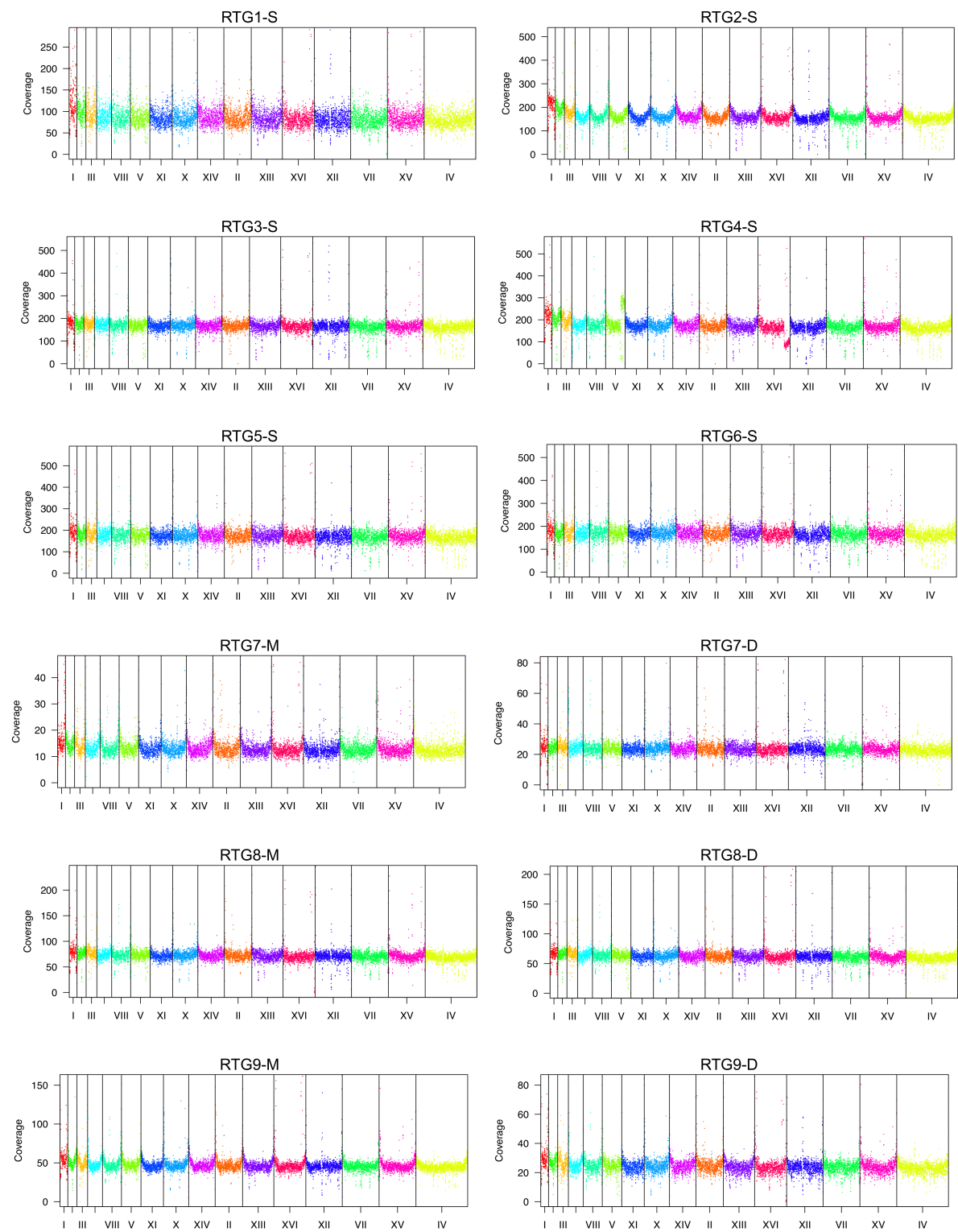

S6 Fig. part 2

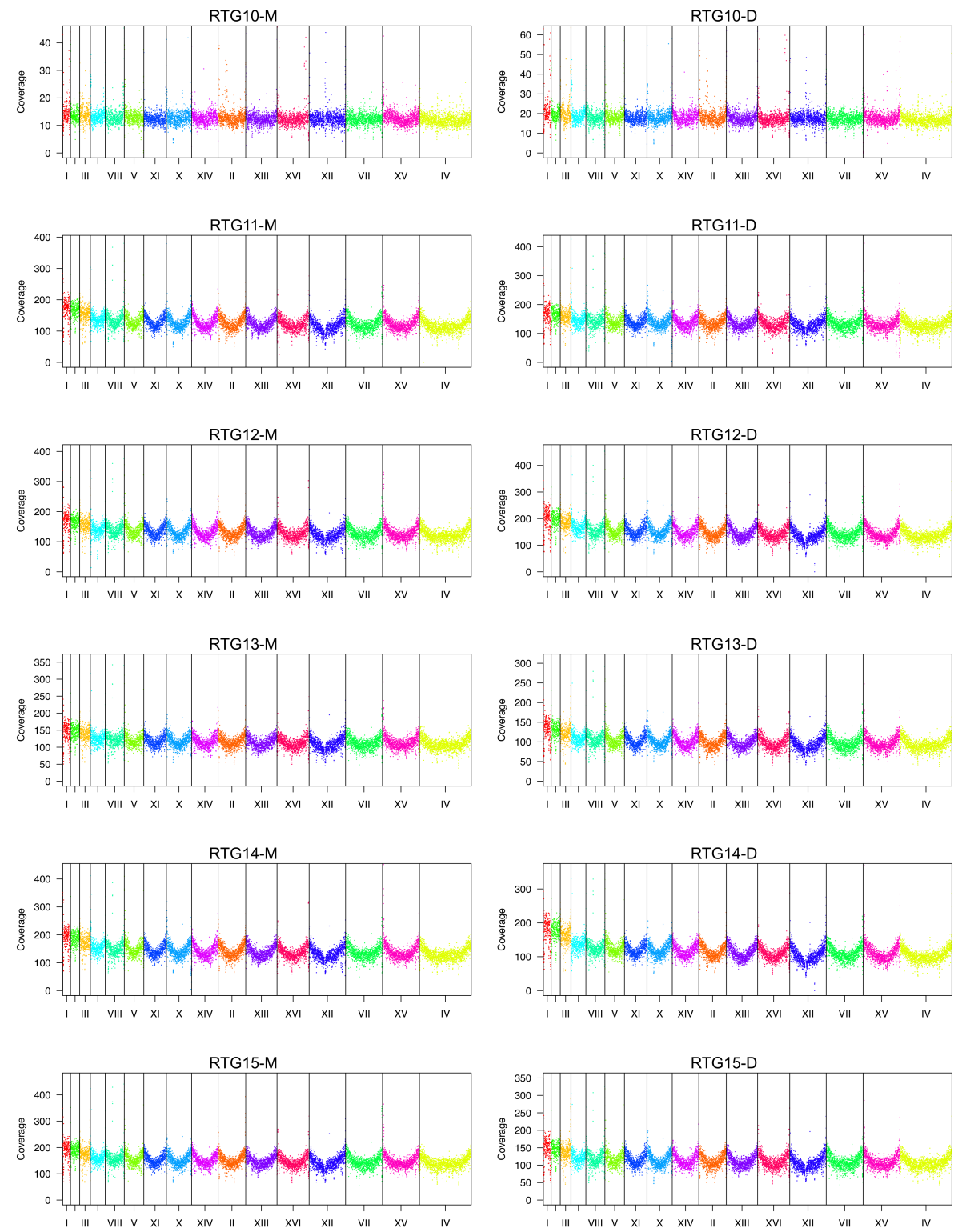

S6 Fig. part 3

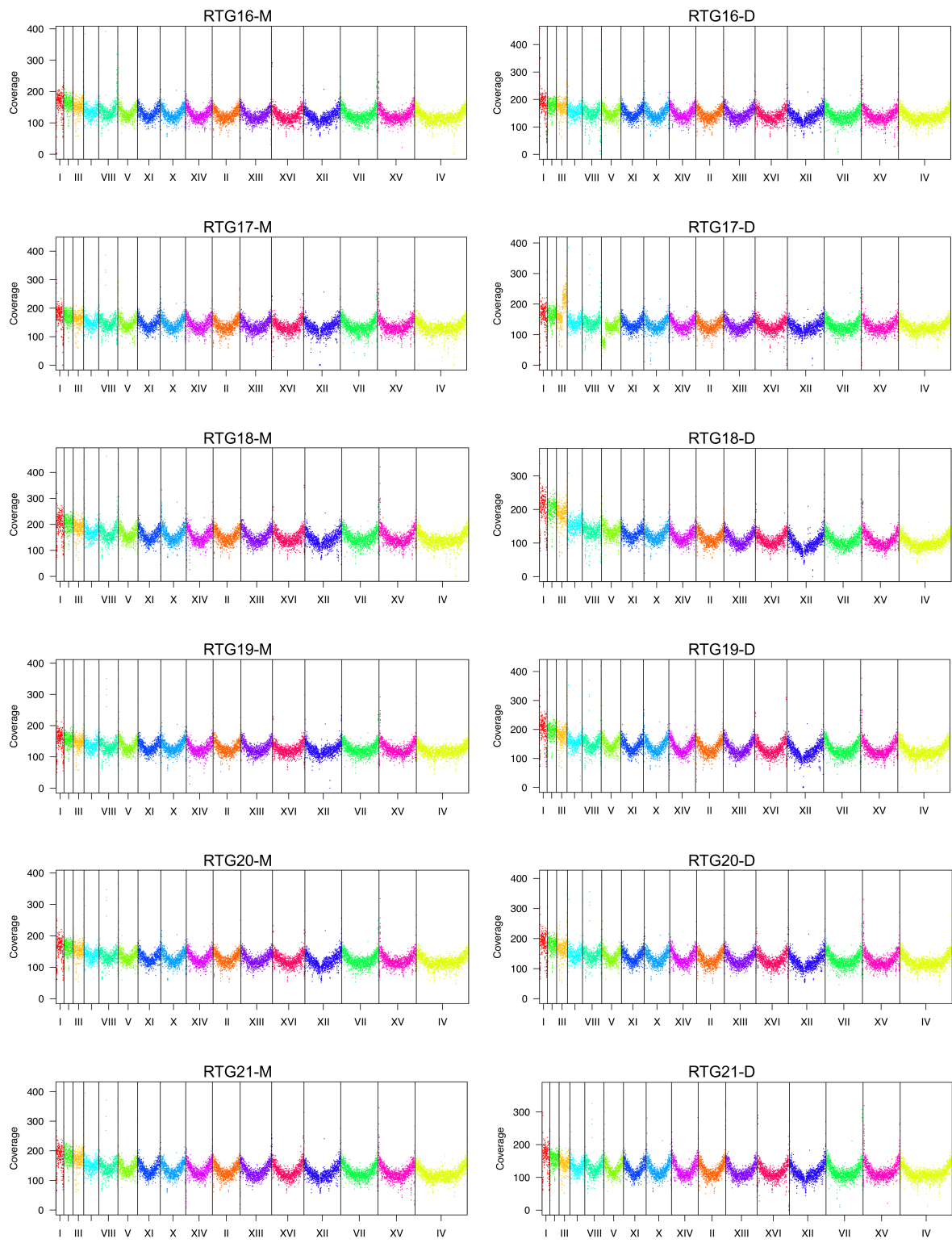

Supplement: S6 Fig — Small chromosomes often exhibits a slightly higher coverage, therefore the visual interpretation of chromosome copy number from the coverage is facilitated when chromosomes are sorted by size. X-axis: The 16 chromosomes are sorted by increasing chromosome size (I, VI, III, IX, VIII, V, XI, X, XIV, II, XIII, XVI, XII, VII, XV, IV). Y-axis: Sequencing read count, averaged per 1-kb window. Note the terminal deletions/duplications in RTG4-S (chromosomes V and XVI) and RTG17-D (chromosomes III and V). (PDF) [file pgen.1005781.s006.pdf]

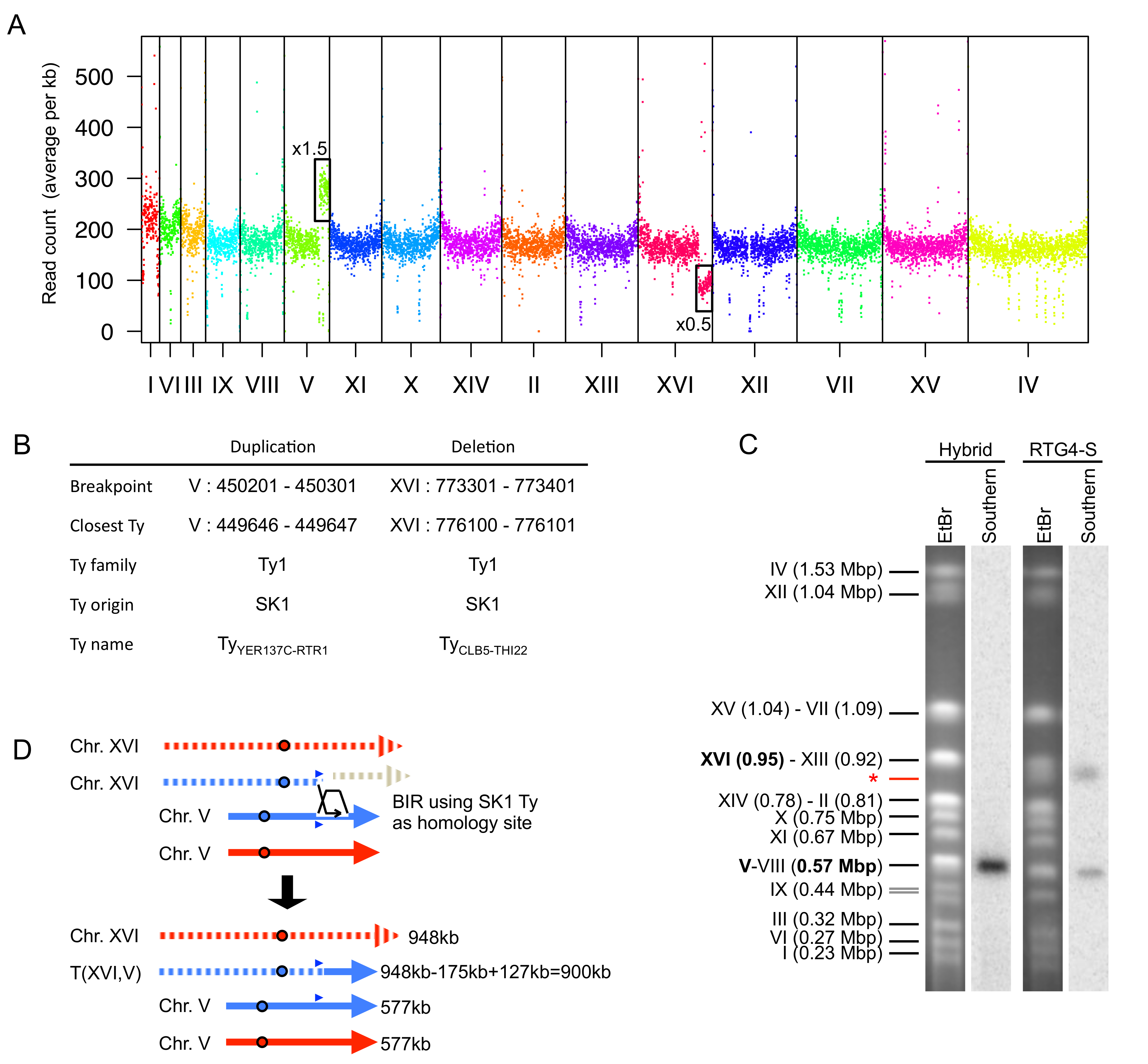

Supplement: S7 Fig — (A) Chromosomal depth coverage of the RTG4-S strain, calculated with a 1kb window. As the cell is diploid, the 1.5 fold change in chromosome V corresponds to a duplication, while the 0.5 fold change on chromosome XVI corresponds to a deletion. (B) Coordinates of the breakpoint for the duplication and deletion in RTG4-S were determined using Control-FREEC software [5], and related to the coordinates of the closest Ty (SGD,[8]). (C) Southern blot analysis of chromosome V. Genomic DNA from the hybrid and RTG4-S strains was extracted in plugs and the chromosomes separated by standard PFGE. Ethidium bromide (EtBr) staining indicates the position of each chromosome on the gel. Chromosome V probe: fragment of the SCC4 gene (coordinate: chromosome V: 462982–464840). The red asterisk indicates the rearranged chromosome V. Instead of a single chromosome V band, in the parental strain, the RTG4-S DNA exhibits an additional band with the size predicted for the chromosome XVI::V non-reciprocal translocation, depicted in panel (D).(D) Ectopic BIR model explaining the gross chromosomal rearrangement that occurred between the Ty1 elements present on the SK1 chromosomes V and XVI. Blue arrowheads: Ty1 elements (from SK1) involved in the BIR. (TIF) [file pgen.1005781.s007.tif]

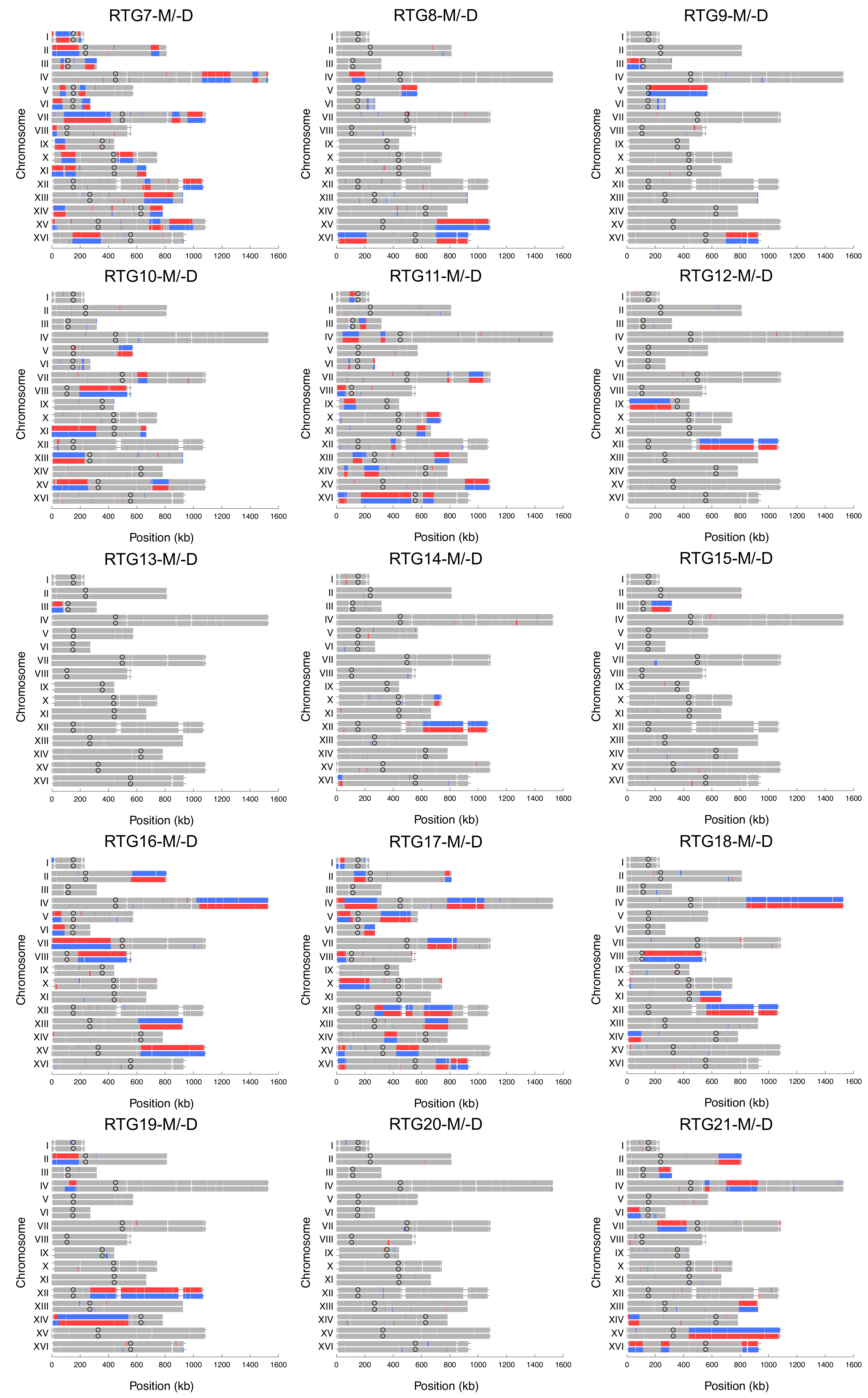

Supplement: S8 Fig — For each chromosome, the RTG-M chromosome is shown on top and the RTG-D chromosome below. (TIF) [file pgen.1005781.s008.tif]

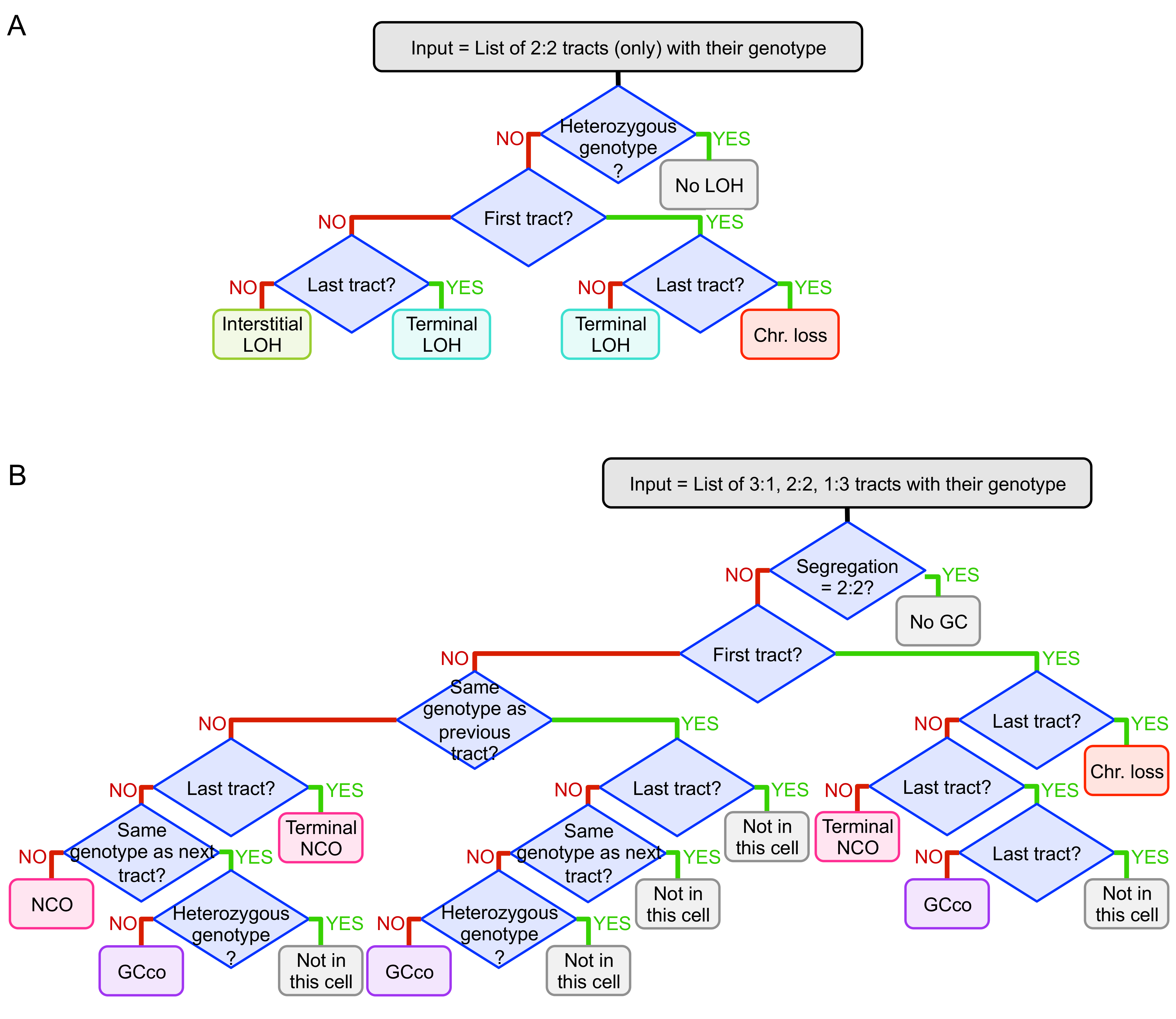

Supplement: S9 Fig — (A) Decision tree allowing the classification of a reciprocal LOH (rLOH) relative to its chromosomal position: a terminal rLOH involves the extremity of one chromosomal arm while an interstitial rLOH is internally located. (B) Decision tree allowing the classification of a non-reciprocal LOH (nrLOH) depending on the relative position of the adjacent rLOH: gene conversions associated with CO are located at the border of the rLOH, while NCO are not contiguous with rLOH. (TIF) [file pgen.1005781.s009.tif]

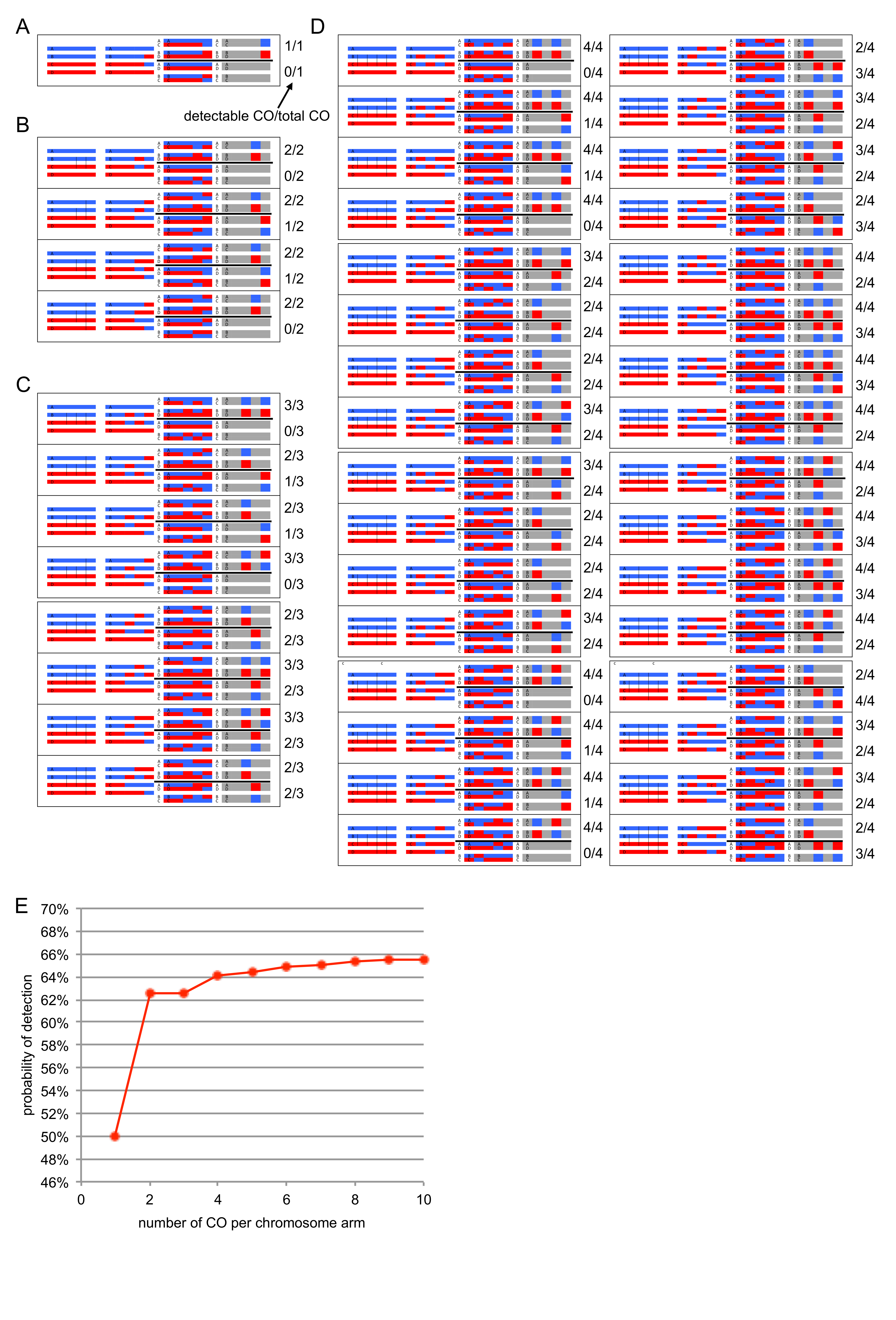

Supplement: S10 Fig — Legends and color code as in Fig 5. (A) Case of 1 CO per chromosomal arm. The numbers adjacent to the panels indicate the ratio of number of detected CO per chromosomal arm versus the total number of CO per chromosomal arm. For example, 0/1 means no LOH is detected albeit one CO occurred. After random sister chromatid segregation, the CO is detected in half of the cases. (B) Case of 2 COs per chromosomal arm. The COs are counted as one when it yields a terminal LOH or two when it yields an interstitial LOH. Globally, the COs are detected in 62.5% of the cases. (C) Case of 3 COs per chromosomal arm. The COs are detected in 62.5% of the cases. (D) Case of 4 COs per chromosomal arm. The COs are detected in 64.1% of the cases. (E) Summary of CO detection per chromosomal arm for 1–10 COs. The detection rate tends to 2/3. (TIF) [file pgen.1005781.s010.tif]

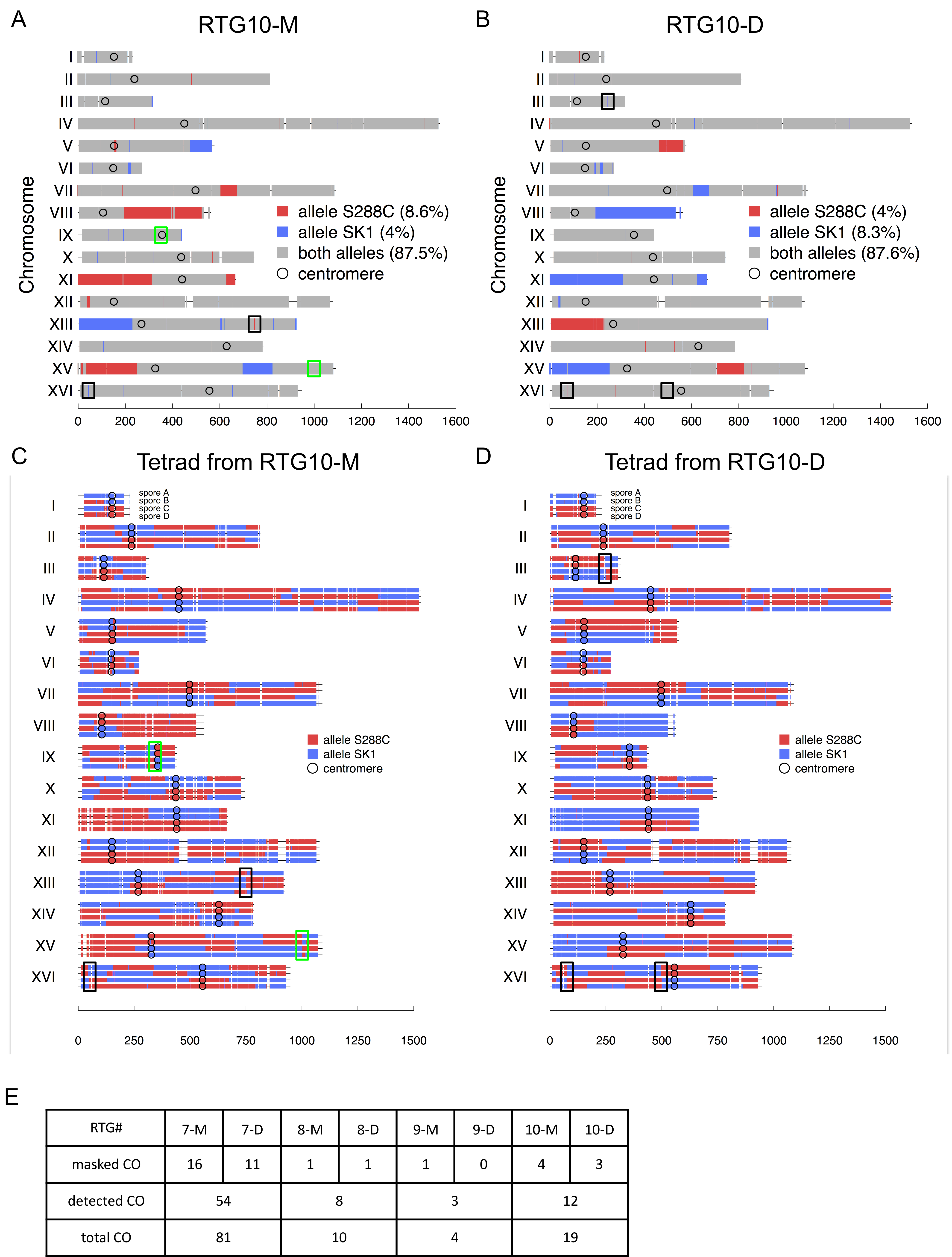

Supplement: S11 Fig — (A-B) Genotype of the RTG10-M and RTG10-D strains, respectively. (C-D) Genotype of 4 spore tetrad issued from the sporulation of the RTG10-M and RTG10-D strains, respectively. The recombination events in the RTG tetrad correspond to the sum of the recombination events that pre-existed in the RTG and the additional recombination events that occurred during the sporulation of the RTG. Note that the regions where the RTG parent was homozygous have the same genotype in the 4 spores. The masked crossovers associated with (black boxes) or without (green box) an adjacent gene conversion segregate as pairs of reciprocal products in the tetrad. Most frequently, the recombination events that occurred during the sporulation of the parent RTG involve only 1 or 2 chromatids. In both tetrads, numerous additional recombination events occurred upon sporulation of the RTG strains, indicating that the RTG retained their capacity to perform meiotic recombination and thus further diversify their genome upon one round of RTG followed by a completed meiosis. (E) Number of masked CO revealed by RTG tetrad sequencing. The number of CO detected by segregation analysis in RTG pairs is given, as well as the total number of CO. (TIF) [file pgen.1005781.s011.tif]

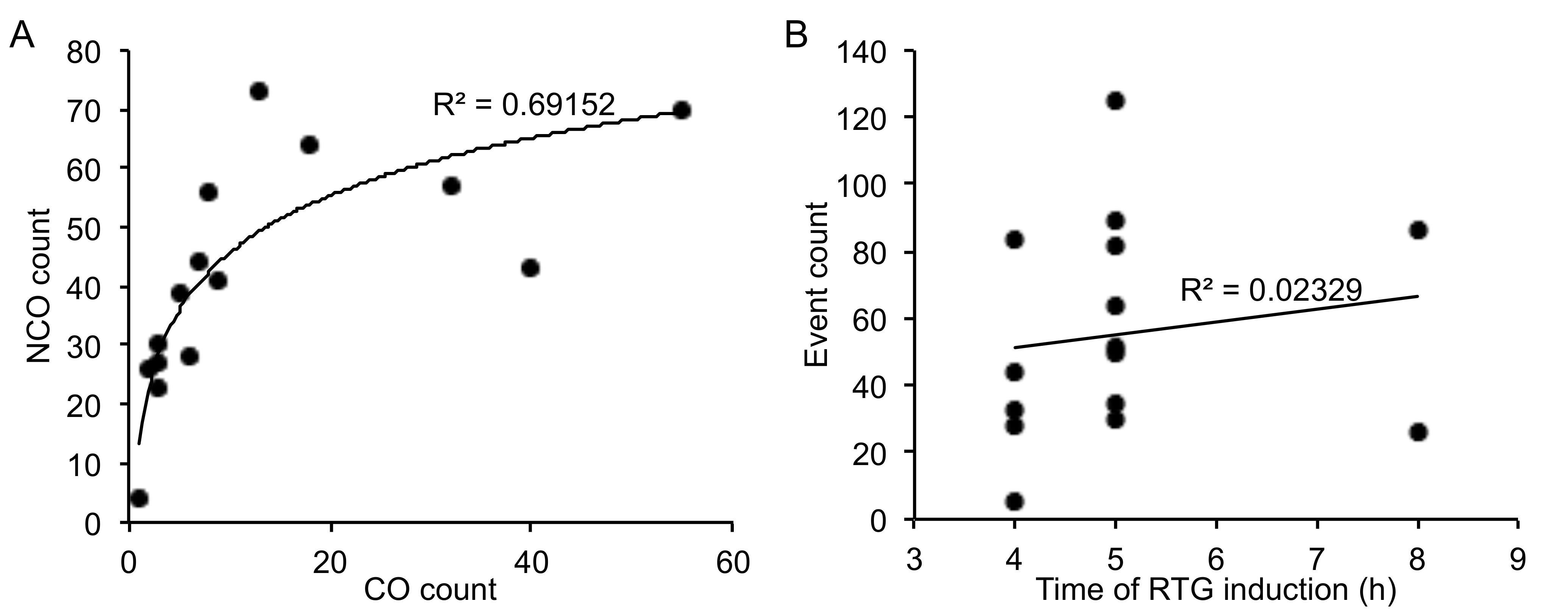

Supplement: S12 Fig — (A) The number of observed NCO per RTG pair correlates with the log10 of the number of COs (logarithmic regression). (B) The total number of observed events (CO+NCO) per RTG pair does not correlate with the time of RTG induction (linear regression). (TIF) [file pgen.1005781.s012.tif]
