# Supplementary material for: Extensive Recombination of a Yeast Diploid Hybrid through Meiotic Reversion
Source: PLoS Genet. 2016 Feb 1;12(2):e1005781. doi: 10.1371/journal.pgen.1005781 (PMC4734685; doi:10.1371/journal.pgen.1005781)
Supplement: S1 Text — (DOCX) [file pgen.1005781.s023.docx]

**Supplementary references**

1. Sun H, Treco D, Schultes NP, Szostak JW. Double-strand breaks at an initiation site for meiotic gene conversion. Nature. 1989;338: 87–90. doi:10.1038/338087a0

2. De Massy B, Nicolas A. The control in cis of the position and the amount of the ARG4 meiotic double-strand break of Saccharomyces cerevisiae. EMBO J. 1993;12: 1459–1466.

3. Li H, Durbin R. Fast and accurate short read alignment with Burrows-Wheeler transform. Bioinformatics. 2009;25: 1754–1760. doi:10.1093/bioinformatics/btp324

4. Quinlan AR, Hall IM. BEDTools: A flexible suite of utilities for comparing genomic features. Bioinformatics. 2010;26: 841–842. doi:10.1093/bioinformatics/btq033

5. Boeva V, Popova T, Bleakley K, Chiche P, Cappo J, Schleiermacher G, et al. Control-FREEC: a tool for assessing copy number and allelic content using next-generation sequencing data. Bioinformatics. 2012;28: 423–425. doi:10.1093/bioinformatics/btr670

6. Anderson CM, Chen SY, Dimon MT, Oke A, DeRisi JL, Fung JC. ReCombine: a suite of programs for detection and analysis of meiotic recombination in whole-genome datasets. PLoS One. 2011;6: e25509. doi:10.1371/journal.pone.0025509

7. Oke A, Anderson CM, Yam P, Fung JC. Controlling meiotic recombinational repair - specifying the roles of ZMMs, Sgs1 and Mus81/Mms4 in crossover formation. PLoS Genet. 2014;10: e1004690. doi:10.1371/journal.pgen.1004690

8. Sasaki M, Tischfield SE, van Overbeek M, Keeney S. Meiotic recombination initiation in and around retrotransposable elements in Saccharomyces cerevisiae. PLoS Genet. 2013;9: e1003732. doi:10.1371/journal.pgen.1003732
